# An All-in-One Sustainable Smartphone Paper Biosensor for Water Toxicity Monitoring Combining Bioluminescence Detection with Artificial Intelligence

**DOI:** 10.1021/acs.analchem.5c02369

**Published:** 2025-08-12

**Authors:** Faisal Nazir, Denise Gregucci, Maria Maddalena Calabretta, Caterina Cambrea, Peyman Vahidi, Stevo Lavrnić, Attilio Toscano, Elisa Michelini

**Affiliations:** † Department of Chemistry “Giacomo Ciamician”, 9296 University of Bologna, Via P. Gobetti 85, 40129 Bologna, Italy; ‡ Department of Agricultural and Food Sciences, University of Bologna, Viale Fanin 50, 40127 Bologna, Italy; § IRCCS Azienda Ospedaliero-Universitaria di Bologna, 40138 Bologna, Italy

## Abstract

Several biosensors for water toxicity monitoring have been reported in the literature; however, none of them fully integrate both analytical and post-analytical steps that are required in a standard laboratory setting before reporting the result. To provide a workflow for smartphone biosensor developers, we implemented a novel procedure that was applied to the standard toxicity assay based on the bioluminescent bacteria *Aliivibrio fischeri*. We addressed the main issues to turn this method into a sustainable all-in-one toxicity paper biosensor, i.e., the immobilization of bacteria, the integration of a calibration curve, and a customized artificial intelligence (AI) application that converts the smartphone picture into user-friendly quantitative information. The biosensor analytical performance was evaluated with different water contaminants and real water samples, showing promising results. A limit of detection of 0.23 ppb was obtained for the cyanotoxin microcystin-LR produced by harmful algal blooms. We also demonstrated for the first time that the inclusion of a calibration curve in a paper sensor, combined with an AI app, enables accurate analyses even when pictures are taken with smartphone models equipped with cameras with different resolutions. To the best of our knowledge, this is the first bioluminescence paper biosensor in which an AI algorithm enables to obtain quantitative results by interpolating the bioluminescent signals from an on-board calibration curve. We believe this novel biosensor will open new opportunities not only for water monitoring, but the same approach could be implemented in any optical smartphone biosensor for applications spanning from onsite analysis to citizen science.

## Graphical abstract

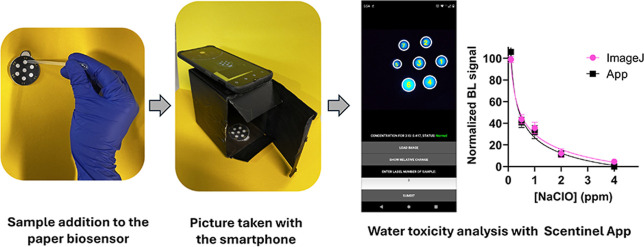

## Introduction

Water monitoring is crucial for ecosystems and human health. Unsustainable practices in industry, agriculture, and urbanization contribute to surface water pollution, releasing harmful chemicals like heavy metals (lead, mercury, cadmium) as well as organic compounds such as polychlorinated biphenyls (PCBs) and toxins derived from harmful algal blooms, posing continuous challenges to both analytical chemists as well as regulators.^1,2^ In addition to agricultural and industrial activities, urban wastewater discharges containing chemicals such as pharmaceuticals and microplastics significantly contribute to water contamination.^3,4^ All of these pollutants pose significant threats to aquatic ecosystems and human health. Therefore, it is of outmost importance to treat these polluted water sources before they are discharged to surface water bodies, especially in the case of water reuse that is becoming vital.^5^ Different water treatment technologies can be implemented, but to evaluate the degree of performance needed and the quality of the final effluent, precise and easy to use measuring instruments are required, possibly at the point of need.^6,7^

Highly sensitive laboratory-based techniques, including liquid chromatography high-resolution mass spectrometry (LC-HRMS) and gas chromatography mass spectrometry (GC-MS), are available to detect several analytes, including polar and nonpolar contaminants of emerging concern (CEC).^8,9^ Nevertheless, not all CECs are detected by these techniques, and the implementation and harmonization of protocols for nontarget screening is required to support regulatory bodies. In addition to conventional screening, biosensors represent a viable solution especially for screening purposes and on-site analysis.^10–12^ The implementation of portable devices integrating biosensors was reported with applications spanning from toxicity monitoring to detection of heavy metals.^13–15^ More promising devices rely on the use of a smartphone as a detector, avoiding the need for hand-held optical detectors. Despite the development of several prototypes, their uptake in the market remains limited.^16,17^ Among the main reasons, there are regulatory issues connected to (i) the on-site use of genetically modified (GM) organisms, (ii) the sustainability and cost of these analytical devices, (iii) the need to perform pipetting or dispensing steps and data elaboration to obtain quantitative results, and (iv) the scarce robustness of smartphone-based devices; i.e., the quality of results is highly dependent on the smartphone camera.

To address these issues, we developed a bioluminescence (BL) sustainable paper sensor relying on *Aliivibrio fischeri* bacteria that provides a novel approach for on-site water analysis. Although several attempts have been reported to implement bacterial bioreporters on portable devices for water monitoring,^18,19^ no complete integration of the entire assay procedure has been achieved. *A. fischeri* strain has the capability of emitting visible light due to the presence of the lux operon encoding both the luciferase (luxAB) and proteins needed for synthesis of the aldehyde substrate (luxCDE).^20^ *A. fischeri* bacteria are widely used for toxicological assessment due to their sensitivity to a broad range of toxic compounds, including metals, organic pollutants, and pesticides. Their luminescence response is highly reproducible, making them ideal for consistent toxicity evaluation. The International Organization for Standardization (ISO) has defined the ISO 11348 method (https://www.iso.org/standard/40518.html), based on *A. fischeri*, for water quality monitoring. This test requires cell culture facilities, benchtop instrumentations, and skilled personnel, increasing the cost and time of the analysis.

To turn this method into a low-cost and user-friendly toxicity biosensor, a bioluminescent paper sensor obtained by entrapping *A. fischeri* has been developed with the goal of implementing both analytical steps (one-step assay) and post-analytical steps (data analysis performed by the Android app) for providing real-time quantitative user-friendly results, in terms of toxicity equivalents, within 15 min. The paper biosensor also integrates a calibration curve, which allows, combined with an AI algorithm, to obtain information on the toxicity directly from a smartphone-captured photo of the sample (Figure 1). This algorithm was implemented into an Android application and the assay was validated with tap and wastewater samples spiked with different contaminants to simulate different conditions such as the presence of disinfectant agent residues, toxins deriving from algae bloom and pesticide residues. The app combined with the paper sensor was also tested with different smartphone models, showing promising results both in terms of analytical performance and ease of use with potential applicability for use by the general population, also in perspective of citizen science.

**Figure 1:**
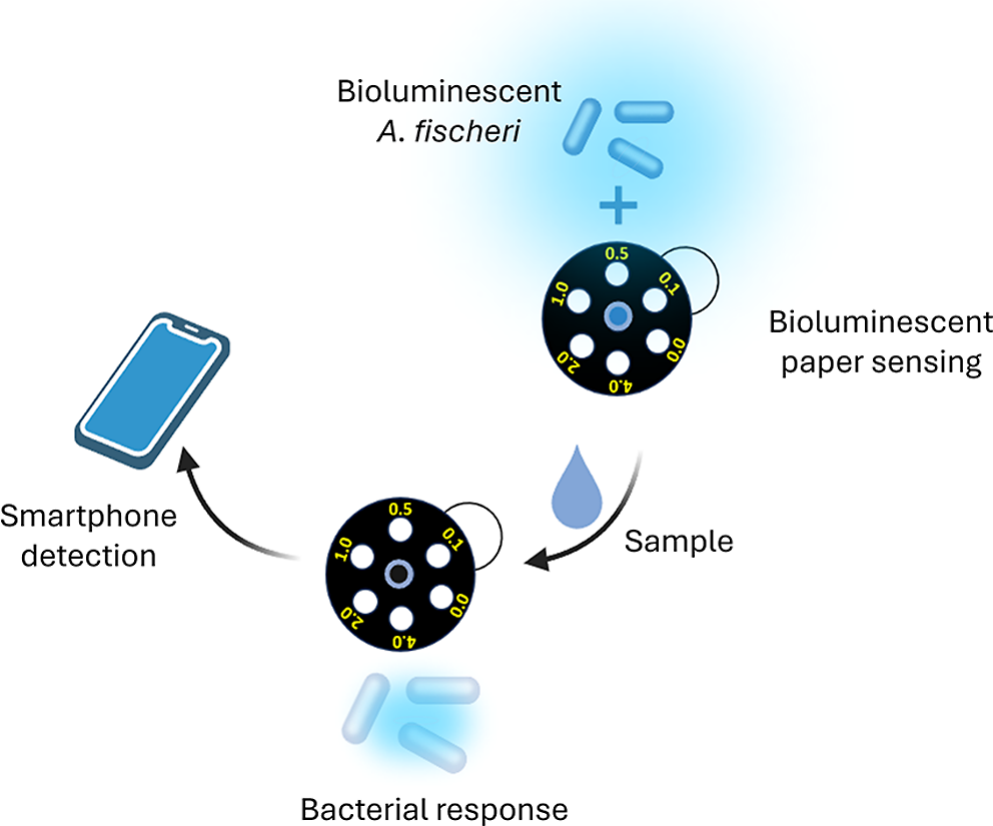
Schematic representation of the paper biosensor principle.

## Materials and Methods

### Chemicals and Reagents

Naturally bioluminescent *A. fischeri* bacteria were kindly gifted by Prof. Stefano Girotti.^20^ NaClO was from commercially available bleach with a declared NaClO percentage of 3.5% _v/v_. Microcystin-LR, 3,5-dichlorophenol, lead nitrate, lysogeny broth (LB) medium, and all reagents for cell culture were from Merck (St. Louis, MO). Whatman 1 CHR cellulose chromatography paper was from GE Healthcare (Chicago, IL, USA) and was used as a support for the design of the bioluminescent sensing paper. OnePlus 6T (OnePlus, Shenzhen, China), Motorola edge 40 neo (Motorola Mobility LLC, Chicago, IL, USA), Huawei P20 (Huawei Technologies Co., Ltd., Shenzhen, GD, China), Samsung Galaxy S20 (Samsung Electronics, Suwon-si, South Korea), and iPhone 12 mini and iPhone 13 mini (Apple Inc., Cupertino, CA, USA) were used for bioluminescent signal acquisitions. Wax printer Phaser 8400 office (Xerox, Norwalk, CT, USA) was used for wax printing.

### Immobilization of *A. fischeri* Bacteria

*A. fischeri* strain was cultured in LB medium with high salinity (10 g/L peptone, 30 g/L NaCl, 5 g/L yeast extract) at 19 °C and with orbital shaking at 140 rpm. Different experimental parameters were evaluated for cell immobilization on paper, including volumes and cell number. An array of 3 × 6 circular hydrophilic wells (7 mm diameter) was designed using PowerPoint software (Microsoft, Redmond, WA, USA) and printed onto W1 paper as a host platform to immobilize *A. fischeri*. The wax-printed paper sensor was then heated at 150 °C for 1 min to allow wax penetration through the paper thickness, effectively forming well-defined hydrophobic boundaries. To prevent leakage during sample addition, the back side of the sensor was sealed with adhesive tape.

Different bacterial concentrations ranging from 1.2 × 10^7^ to 2.0 × 10^7^ cells/well and supplements (trehalose and glycerol) were tested for *A. fischeri* entrapment in agarose-based hydrogels (see Supporting Information).

In optimized conditions, the BL paper sensor was obtained by entrapping *A. fischeri* (cell suspension with OD600 = 5.0 in LB medium) in a 0.5% _w/v_ agarose hydrogel matrix. A 3% _w/v_ agarose hydrogel was first prepared in sterile Milli-Q water by heating, then, when the agarose hydrogel reached a temperature of around 60 °C, an 80 μL volume was added to 420 μL of *A. fischeri* suspension in LB medium (final temperature of about 30 °C) (see Heat Conduction Calculation in Supporting Information). A volume of 20 μL of the bacterial suspension–agarose was deposited immediately into each well. Then, the wells were equilibrated at room temperature (25 °C) for 30 min before performing the analysis.

### Real-Sample Analysis

The suitability of the BL sensing paper was tested with real water samples, including six tap water and six industrial wastewater samples (provided by a carwash activity) spiked with different concentrations of the model analytes (NaClO from 0.1 to 4.0 ppm, microcystin-LR from 1.5 to 40 ppb, 3,5-dichlorophenol from 1.0 to 6.0 ppm, and lead nitrate from 5.0 to 100 ppb) and analyzed following the assay procedure described above. Since their viability is affected by toxic agents, the decrease in BL was used to assess the toxicity of samples. BL signals were normalized with respect to the control. Each sample was tested in triplicate, and experiments were repeated with three different paper sensors.

### Design of the Toxicity Sensing Paper and NaClO Toxicity Assay

To obtain ready-to-use portable sensing paper for toxicity assessment of water samples, a circular flower-like paper (30.0 mm diameter) has been created by wax printing technology using a Phaser 8400 office wax printer (Xerox, Norwalk, CT, USA). The toxicity sensing paper was designed to contain seven hydrophilic wells (diameter of 5 mm each) to immobilize the BL bacteria: six external wells for the calibration curve (S0, S1, S2, S3, S4, and S5) and a central well for the sample. The optimized procedure requires (i) dispensing of a 30 μL-volume of standard solutions and sample, (ii) incubation from 1 to 15 min at room temperature, (iii) placement of the paper sensor in the cardboard dark box (8.5 × 11.5 × 10.0 cm) to avoid external light interference, and (iv) acquisition with the OnePlus 6 smartphone camera (30 s integration time, ISO1600). Images were analyzed in parallel with ImageJ software and the Android-based application.

### Android Application Development

An Android app, called *Scentinel*, was developed in Python using the Kivy framework library to perform image analysis within an integrated development environment (IDE), focusing on code validation, statistical analysis through curve fitting, and adjustments of AI parameters. Then, the program was converted into an Android app by finetuning the probability parameter and nonmaximum suppression (NMS) for the AI model to 0.67 and 0.01, respectively, to increase the confidence level for the prediction of the BL signal and filtering low level of signals.

Figure 2 illustrates the user interface of the *Scentinel* application and its functionality. Initially, the image of the paper sensor was uploaded to the *Scentinel* app and transferred to the Microsoft Azure server. On this server, image processing tasks such as instance segmentation and feature extraction were performed. The processed and labeled image along with the extracted features were sent back to the *Scentinel* app (Figure 2a). The OpenCV library was used to label each region of interest (ROI) and extract the corresponding BL signal value. All details are provided in the Supporting Information section.

**Figure 2:**
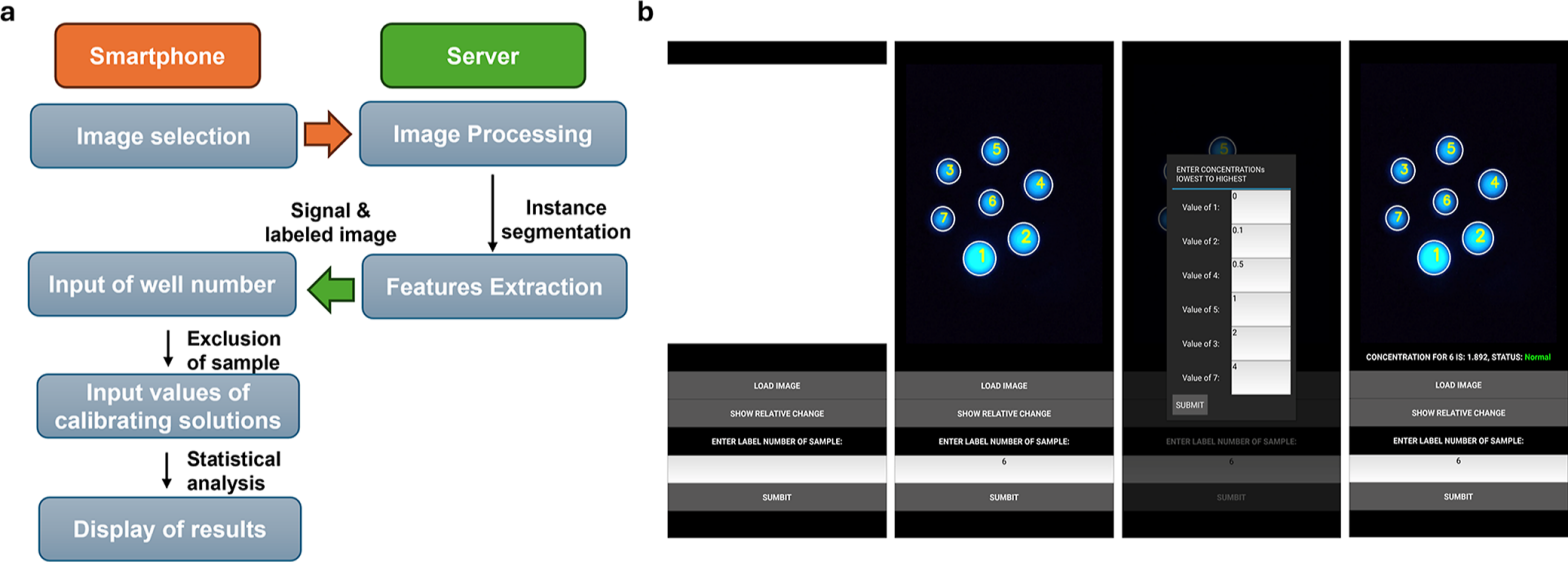
(a) Representation of services handled by the smartphone and server. (b) Screenshots of application graphical user interphase (GUI) representing buttons, input prompts, image, and concentration display.

### Smartphone Acquisitions and Statistical Analysis

For optimization, a OnePlus 6 smartphone secondary sensor (20 MP Sony Exmor RS IMX 376 K, BSI CMOS color sensor with 1.0 μm pixels, *f*/1.7 aperture) was used with an ISO 1600 and 30 s integration time. All pictures were analyzed with ImageJ (v. 1.53 k, National Institutes of Health, Bethesda, MD, USA), and the BL signal intensities, expressed as relative light units (RLUs), were quantified over the ROI defined in correspondence with the sensor wells. GraphPad Prism v.8 software (GraphPad Software, LaJolla, CA, USA) was used for data elaboration. The limit of detection (LOD) was calculated as the mean value of the blank minus three times the standard deviation. The limit of quantification (LOQ) was calculated as the mean value of the blank minus ten times the standard deviation. All measurements were performed in triplicate and repeated at least three times.

### Analytical Performance of Different Smartphones and Data Elaboration with *Scentinel* Application

To evaluate the analytical performance of different smartphone-integrated CMOS sensors and to compare data elaboration obtained with ImageJ software and *Scentinel* application, BL images of toxicity sensing papers (3 × 6 wells, 7 mm diameter) incubated with 50 μL of NaClO (0.0–4.0 ppm) for 1 min at room temperature (25 ± 2 °C) were acquired using five different smartphones: Motorola edge 40 neo (16 s acquisition time, ISO 1600), Samsung Galaxy S20 (30 s acquisition time, ISO 1600), Huawei P10 (8 s acquisition time, ISO 1600), iPhone 12 mini (10 s acquisition time, night mode), and iPhone 13 mini (20 s acquisition time, night mode).

### Stability and Reproducibility Studies

Biosensor stability and reproducibility were evaluated over a 14 day period by storing the biosensors at 4 °C in Petri dishes sealed with Parafilm tape. BL intensities were normalized with respect to the BL signal obtained from freshly immobilized *A. fischeri* at day 0. At the same time, reproducibility studies were performed in triplicate by incubating the paper-toxicity biosensor with 1 ppm of NaClO for 1 min at room temperature. BL intensities obtained in the sample wells were normalized to BL data in the control wells. Images were acquired with a OnePlus 6 smartphone (30 s, ISO 1600) and analyzed with ImageJ. All measurements were performed in triplicate and repeated at least three times with different toxicity paper-based biosensors.

## Results and Discussion

Several biosensors for monitoring water toxicity have been reported; however, none fully incorporate all the essential analytical and post-analytical steps typically performed in a standard analytical laboratory before delivering results. We addressed all the needs required to obtain a robust all-in-one biosensor, which only requires sample addition and a photo taken with the smartphone. As a proof of concept, we applied this workflow to the standard toxicity bioassay based on bioluminescent *A. fischeri* bacteria. Our approach tackles key challenges in transforming an analytical method, the ISO 11348 method “Water quality—Determination of the inhibitory effect of water samples on the light emission of *Vibrio fischeri* (Luminescent bacteria test)”, into a sustainable, all-in-one toxicity biosensor. Since the biosensor is not selective, we first investigated its suitability for detecting target key contaminants with the required sensitivity. In particular, we tested toxins produced by cyanobacteria, residues of disinfectants, pesticide residues, and heavy metals. The frequency of extreme weather events is causing concerns about harmful cyanobacterial algal blooms producing highly toxic metabolites, including microcystin-LR.^21^ Another target analyte is 3,5-dichlorophenol, a metabolite of polychlorinated phenols (i.e., Pestanal) and benzene hexachloride pesticides.^22^ As a model heavy metal, lead (Pb) was chosen due to frequent exposures from lead-containing plumbing and severe health effects including high neurotoxicity, especially in children.^23^

### Optimization of *A. fischeri* Immobilization

Preliminary experiments were performed to optimize *A. fischeri* immobilization on paper. Agarose-based hydrogels were selected using different cell concentrations as previously reported^24^ with slight modifications. *A. fischeri* cells with an OD_600_ of 5.0 were entrapped in 0.25% _w/v_ agarose +10% _w/v_ trehalose hydrogel and deposited on paper. Different bacterial concentrations were tested (range 1.2 × 10^7^–2.0 × 10^7^ cells/well); a 5.2% decreased bioluminescence signal was obtained using the lowest concentration, while the highest number of cells resulted in a 26.1% signal increase compared to the same cell suspension in liquid culture. BL intensities of entrapped *A. fischeri* cells (OD_600_ 5.0) were evaluated using different hydrogel compositions with 0.5% _w/v_ agarose, 0.25% _w/v_ agarose +15% _w/v_ glycerol, and 0.25% _w/v_ agarose +10% _w/v_ trehalose (Figure S1), showing increased BL intensities (+28.2%) for both 0.5% _w/v_ agarose and 0.25% _w/v_ agarose +10% _w/v_ trehalose, and a 4.1% increase for the hydrogel composed by 0.25% _w/v_ agarose and 15% _w/v_ glycerol.

### Toxicity Test Optimization with the Model Analyte

After these preliminary evaluations, the hydrogel composition, incubation times, and volume of samples were investigated to identify the suitable combination providing active bacterial cells on paper and the highest sensitivity of the toxicity paper sensor. The analytical performance of the toxicity paper biosensor was assessed by using four environmental contaminants as model analytes: NaClO, microcystin-LR, 3,5-dichlorophenol, and lead nitrate. For each analyte, the incubation time (1, 15, and 30 min) and the volume of the sample (30 and 50 μL) were evaluated with cell suspension (OD_600_ 5.0) in 0.5% _w/v_ agarose and 0.25% _w/v_ agarose +10% _w/v_ trehalose. As expected, BL signals obtained in the presence of an analyte that causes immediate toxicity, such as NaClO, did not significantly differ using the two hydrogel compositions (Figure S2). This confirms that for NaClO, both entrapment methods represent a viable solution. Incubation times exceeding 15 min result in a complete loss of bioluminescence, even at the lowest tested NaClO concentration (Figure S3), confirming that for chemicals causing an immediate cell death, images must be acquired 1 min after sample addition.

When bacteria entrapped in the trehalose-containing hydrogel were incubated with 3,5-DCP (0.0, 2.0, 3.5, and 6.0 ppm), bioluminescence signals acquired at min 1 showed a negligible inhibitory effect (0.7% decrease in the presence of 2.0 ppm 3,5-DCP) with no concentration-dependent effect (Figure S4). After 30 min of incubation, the decrease in the BL signal was not proportional to 3,5-DCP concentration increase with a nonsignificant decrease in BL (i.e., 99.0% of the signal with 6.0 ppm 3,5-DCP). This effect could be ascribed to the presence of trehalose, which is known to protect bacteria under stress conditions, as previously reported for plants and bacteria.^25^ Bacteria entrapped in 0.5% _w/v_ agarose (without trehalose) showed a significant decrease in the BL signal when incubated with increasing concentrations of 3,5-DCP with a 11.0% decrease in the BL signal after 15 min incubation with 6.0 ppm 3,5-DCP (Figure S4a). A similar behavior was observed with MC-LR, which did not produce an immediate toxicity effect on bacteria. In the presence of 40.0 ppb MC-LR, a 16% decrease of the BL signal was observed with agarose-entrapped bacteria, while only 4% decrease was observed with bacteria entrapped with the 0.25% _w/v_ + trehalose 10% _w/v_ hydrogel (Figure S5a,b). We evaluated the hydrogel composition effect in the presence of a heavy metal, i.e., lead, which can leach into water from pipes. No concentration-dependent effect was observed in both the two hydrogel matrices, with only a 7, 8, and 9% signal decrease obtained with bacteria entrapped in the 0.25% _w/v_ + trehalose 10% _w/v_ hydrogel after 30 min incubation time with 5, 15, and 75 ppb of lead nitrate, respectively (Figure S6). For the other incubation times, however, the same behavior observed with MC-LR was observed. Consequently, the initial hypothesis that trehalose could interfere with the biosensor response due to its nutrient and protective action was confirmed. In optimized conditions, the BL paper sensing was obtained with cell suspension (OD_600_ 5.0) entrapped in the 3.0% _w/v_ agarose hydrogel (final agarose concentration 0.5%_w/v_). Different sample volumes were tested (30 and 50 μL). BL signals were acquired after 1 min for NaClO (Figure S7) and after 15 min for microcystin-LR (Figure S8), 3,5 dichlorophenol (Figure S9), and lead nitrate (Figure S10). The 50 μL sample volume was selected because it allowed better discrimination between the blank and analyte-containing samples. This volume offered the best compromise to enable BL signal detection via the smartphone camera with the required sensitivity.

### Analytical Performance of the Toxicity Sensing Paper

In optimized conditions, the toxicity sensing paper is prepared by adding a 20 μL volume of the bacterial suspension–agarose to each well; then, the paper sensor is equilibrated at room temperature (25 °C) for 10 min. To perform the toxicity test, a volume of 50 μL of either the sample or standard analyte is added to the paper sensor and a picture is taken at 1 or 15 min, depending on the analyte toxicity. Because no substrates are required, this poses no inconvenience to the end-user, who can simply capture images at both time points. With the optimized method, toxicity dose–response curves were obtained by using the sensing paper for the analysis of NaClO (from 0.1 to 4.0 ppm), microcystin-LR (from 1.5 to 40 ppb), 3,5-dichlorophenol (from 1.0 to 6.0 ppm), and lead nitrate (from 5.0 to 100 ppb) (Figure 3). Calibration curves were obtained with ImageJ software and with the *Scentinel* app, and a coefficient of determination of 0.990 or higher was obtained for all cases. The LODs and LOQs for NaClO were 0.11 and 0.15 ppm, respectively, for ImageJ, and 0.17 and 0.58 ppm for the *Scentinel* app (Figure 3a), respectively. These results confirm the suitability of the toxicity paper sensor to measure the maximum allowed concentration of free residual chlorine in drinking water of 4 and 5 ppm fixed by the U.S. Environmental Protection Agency (EPA) and the World Health Organization (WHO), respectively. Concerning microcystin-LR, for which the maximum allowed concentration in waters for human consumption is fixed at 1.0 ppb by the WHO (WHO/HEP/ECH/WSH/2020.6), LODs of 0.65 and 0.23 ppb were obtained with ImageJ analysis and *Scentinel* app, respectively; LOQs were 8.55 and 26.5 ppb, respectively (Figure 3b). For the 3,5-dichlorophenol, LODs of 4.50 and 4.93 ppm and LOQs of 6.76 and 5.12 ppm were obtained with ImageJ analysis and the *Scentinel* app, respectively. In this case, due to a lower toxicity of 3,5-DCP, the sensor analytical performance does not fulfill the required regulatory limits (e.g., the WHO limit for 3,5-DCP in drinking water is 0.5 ppm); however, the toxicity sensor is not specific and is intended for the first screening assay (Figure 3c). When a sample contains multiple contaminants, potentially having additive or synergistic effects on total toxicity, the sensor would provide the total effect of the sample; therefore, we believe this is not a shortcoming of the method. LODs for lead nitrate were 74.5 and 5.0 ppb, and the LOQs were 71.4 and 26.2 ppb, obtained with ImageJ analysis and the *Scentine*l app, respectively (Figure 3d), supporting the potential use of the application for the analysis of lead in drinking water (maximum allowed concentration according to the WHO is 10 ppb). According to a recent study, several commercial kits for lead analysis were not able to detect such levels of lead since the detection limits were in the order of 10–20 mg/L.^26^ We compared these results to a recently reported biosensor in which *A. fischeri* was immobilized on a 96-well plate with two strategies, either agar or graphene oxide, and the bioluminescence signal was read with a smartphone.^27^ Despite the promising results obtained by Bergua et al., full integration of the *A. fischeri* in a portable device was not reported, and the limits of detection for the tested pesticides (from milligrams per liter to μg per liter) were still not adequate to those requested by the regulatory agencies (ng/L range). Our smartphone-based biosensor offers several advantages over traditional *A. fischeri*-based toxicity assays such as Microtox and DeltaTox systems, which are either laboratory-based assays or transportable systems requiring power supply and pipetting steps with skilled personnel. In contrast to the Microtox assay, which requires a temperature of 15 °C to rehydrate freeze-dried bacteria and conduct the analysis, our biosensor operates effectively at 25 °C while maintaining satisfactory analytical performance. Moreover, in terms of cost-effectiveness, these conventional approaches can be prohibitively expensive because of the instrument costs and maintenance, whereas our method is based on a low-cost paper sensor and a smartphone, making it accessible to a wider range of customers.^28^ Traditional systems often require trained personnel and laboratory facilities, increasing the cost and time of the analysis. Thanks to an analysis via image capture and AI-based processing, our system can perform real-time analysis and provides instant feedback. In addition, AI integration ensures automated and consistent data interpretation,^29^ reducing human error and enhancing reproducibility.

**Figure 3:**
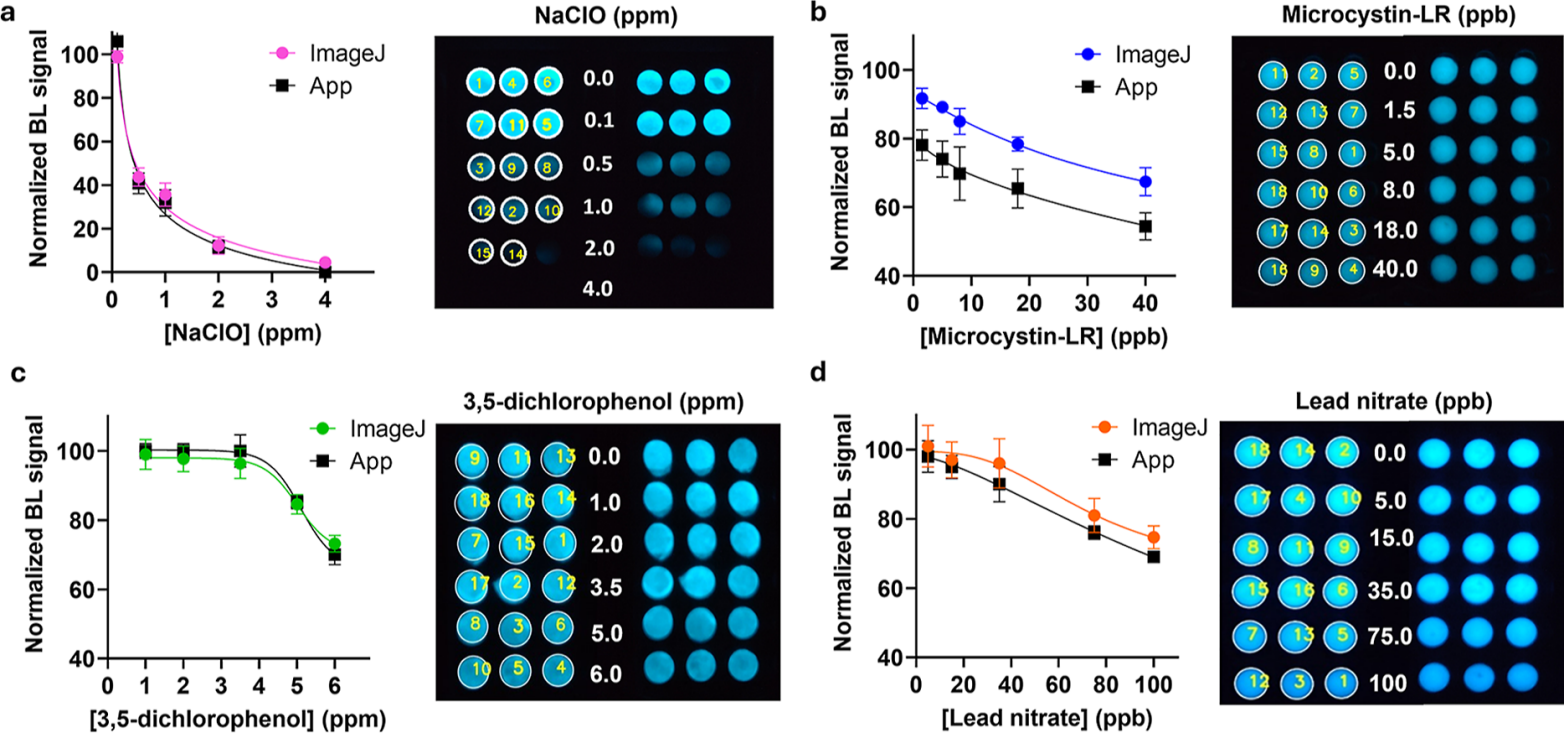
Dose–response curves using BL signals obtained with the OnePlus6 smartphone camera (30 s integration time, ISO 1600) and quantified by ImageJ software (colored) and AI-assisted application (black) for (a) NaClO in distilled H_2_O (1 min incubation t time), (b) microcystin-LR in EtOH 5% _v/v_ (15 min incubation time), (c) lead nitrate distilled H_2_O (15 min incubation time), (d) 3,5-dichlorophenol EtOH 5% v/v (15 min incubation time).

### Real Samples and Recovery Studies

To evaluate the analytical performance of the paper biosensor and investigate the potential matrix effects, real water samples of tap water and wastewaters were spiked with NaClO (Figure S11), microcystin-LR, 3,5-dichlorophenol, and lead nitrate at different concentrations. Toxicity tests were performed with the optimized assay procedure, and data extractions were obtained from the analysis of the bioluminescent signals with ImageJ software and the *Scentinel* App (Figure 4). The toxicity observed in the presence of the four analytes in tap and wastewaters reflects that obtained in dH_2_O for both ImageJ and the App analysis. Differences in the inhibition profile of bacteria are due to the complexity of the matrices. Recovery values for microcystin-LR, 3,5-dichlorophenol, and lead nitrate were in the range 91–120% for drinking waters (Table S1) and 92–128% for wastewaters (Table S2), while, as expected, the acute toxicity of NaClO yielded lower recoveries.

**Figure 4:**
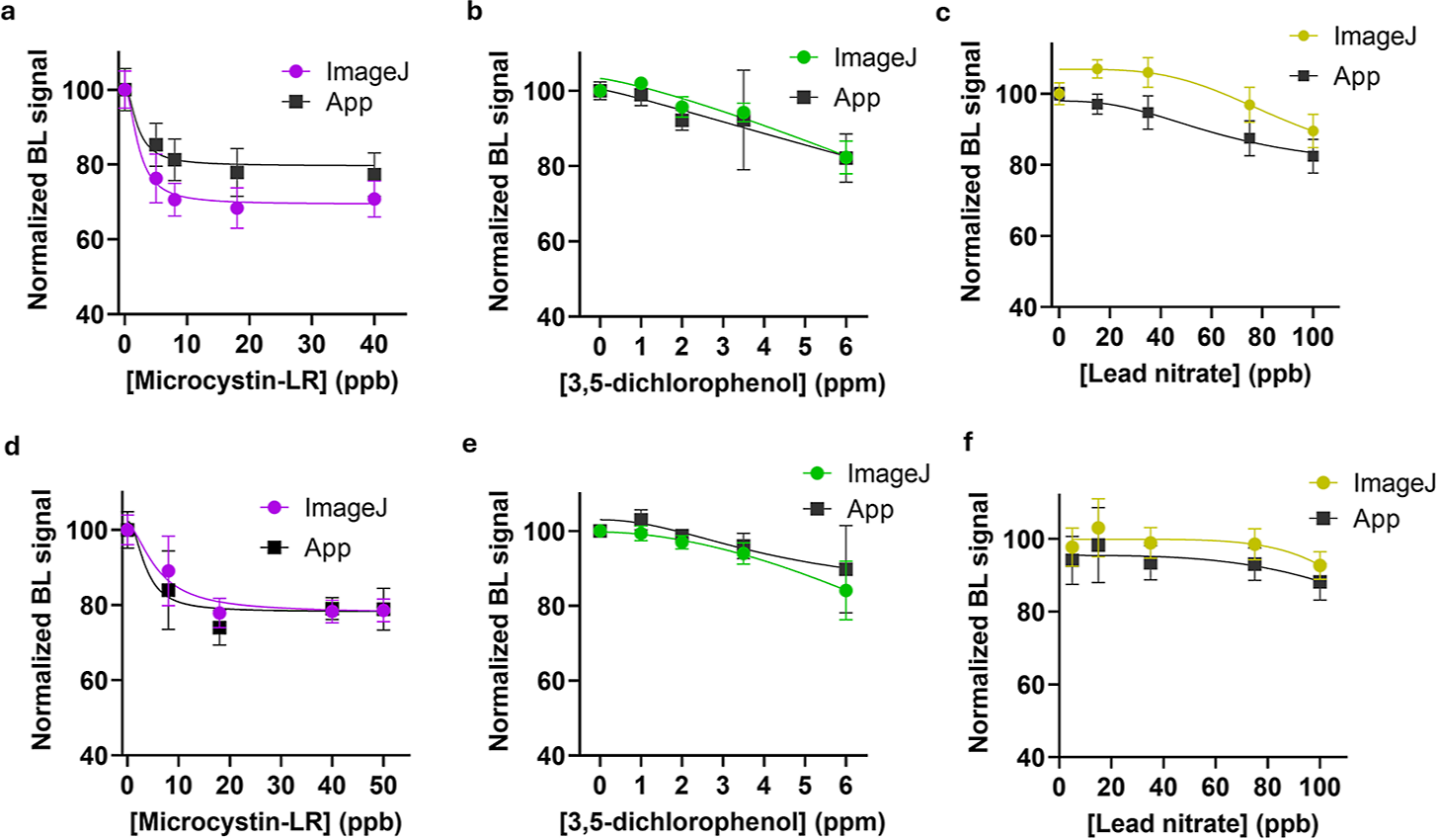
Dose–response curves in (a–c) drinking water and (d–f) in wastewaters obtained with the paper-based biosensor. BL signals were acquired with a OnePlus6 smartphone camera (30 s integration time, ISO 1600) and quantified by ImageJ software (colored) and the AI-assisted application (black) for (a,d) microcystin-LR (15 min incubation time), (b,e) 3,5-dichlorophenol (15 min incubation time), (c,f) lead nitrate (15 min incubation time).

### Data Elaboration with the AI *Scentinel* App Using Different Smartphones

One of the main limitations of smartphone biosensors is the high variability of the response due to the different sensitivities of smartphone-integrated CMOS.^30,31^

Image acquisition from different mobile phones is affected by different sensitivities of sensors and processing algorithms limiting the accuracy of analytical procedures and applicability of Machine Learning (ML) algorithms.^30,31^ To address this issue, we designed a BL toxicity paper sensing which includes an on-board calibration curve enabling the analysis of standard solutions as well as the unknown sample; this strategy avoids artifacts due to signal variations derived from noncontrolled environmental conditions (e.g., temperature, humidity). We evaluated our approach by capturing images of toxicity paper biosensors (three biosensors for each smartphone analysis) incubated with different concentrations of NaClO with various mobile phones, from flagship to low-tech models. For iOS smartphones, the picture was first taken with the iOS smartphones and then transferred to an Android smartphone with the *Scentine*l app installed. Pictures of the paper toxicity biosensor incubated with different concentrations of NaClO (range 0.0–4.0 ppm) were taken with five smartphones (Motorola edge 40 neo, Samsung Galaxy S20, Huawei P10, iPhone 12 mini, and iPhone 13 mini) and compared with those obtained with the OnePlus6. All the tested mobile phone cameras successfully allowed the retrieval of quantitative information with decent dose–response curves obtained with ImageJ analysis (Figure 5a) or the *Scentinel* App (Figure 5b). Motorola edge 40 neo provided the lowest LOD with 0.06 ppm for NaClO obtained both with image J analysis and the *Scentinel* app (Table S3 shows LODs obtained with the different smartphones).

**Figure 5:**
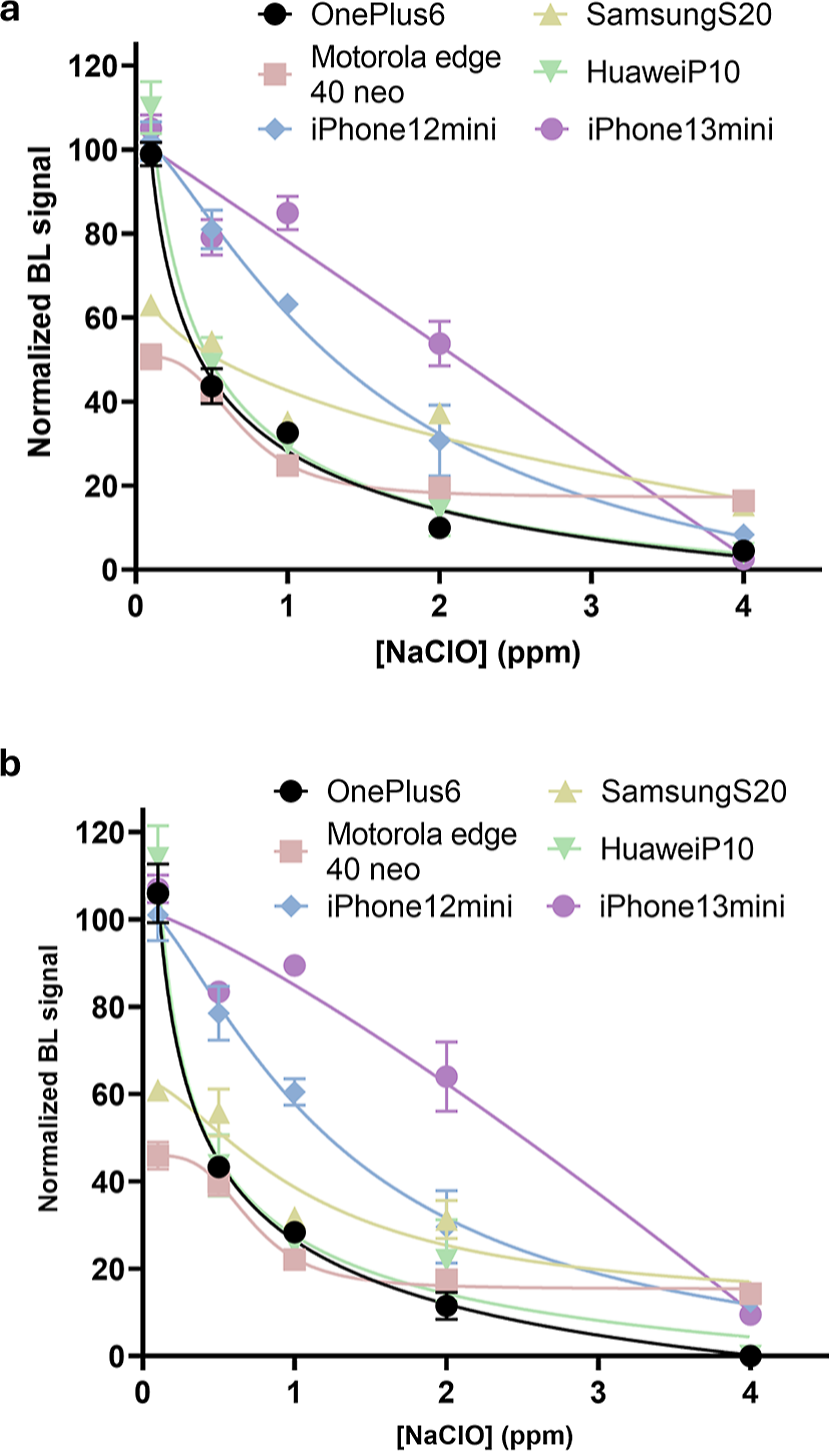
NaClO toxicity curves obtained in ddH_2_O, by incubating for 1 min the paper toxicity biosensor with NaClO (from 0.0 to 4.0 ppm) and acquiring BL signals with different smartphones (OnePlus 6, Motorola edge 40 neo, Samsung S20, HuaweiP10, iPhone 12 mini, and iPhone 13 mini). BL intensities were quantified by using (a) ImageJ software and the (b) *Scentinel* app.

Furthermore, our application is compatible with all Android-based mobiles, enabling quantification even with lower ISO ranges and acquisition times. To the best of our knowledge, this is the first bioluminescence paper biosensor in which an AI algorithm enables to obtain quantitative results by interpolating the bioluminescent signals from an on-board calibration curve. Since the biosensor is not selective for specific analytes, the read-out provides both quantitative and qualitative information, the latter in terms of general warning, for example, “Toxic” and “Safe”, these parameters have been preliminary set according to a predefined level of toxicity caused by the sample and can be modified according to the needs. This application could have a more general use for all optical biosensors, including those with colorimetric and fluorescence output, for turning a qualitative response into quantitative information, without the need for separate data elaboration.

### Design of the Flower-like Paper Toxicity Biosensor

After characterization of the biosensor analytical performance, we designed a circular flower-like paper sensor containing six external wells for the calibration curve with standard solutions of the analyte and a central well for the sample test (Figure 6a). For easy handling, a 1.5 cm paper grip has been added to the sensor (Figure S12).

**Figure 6:**
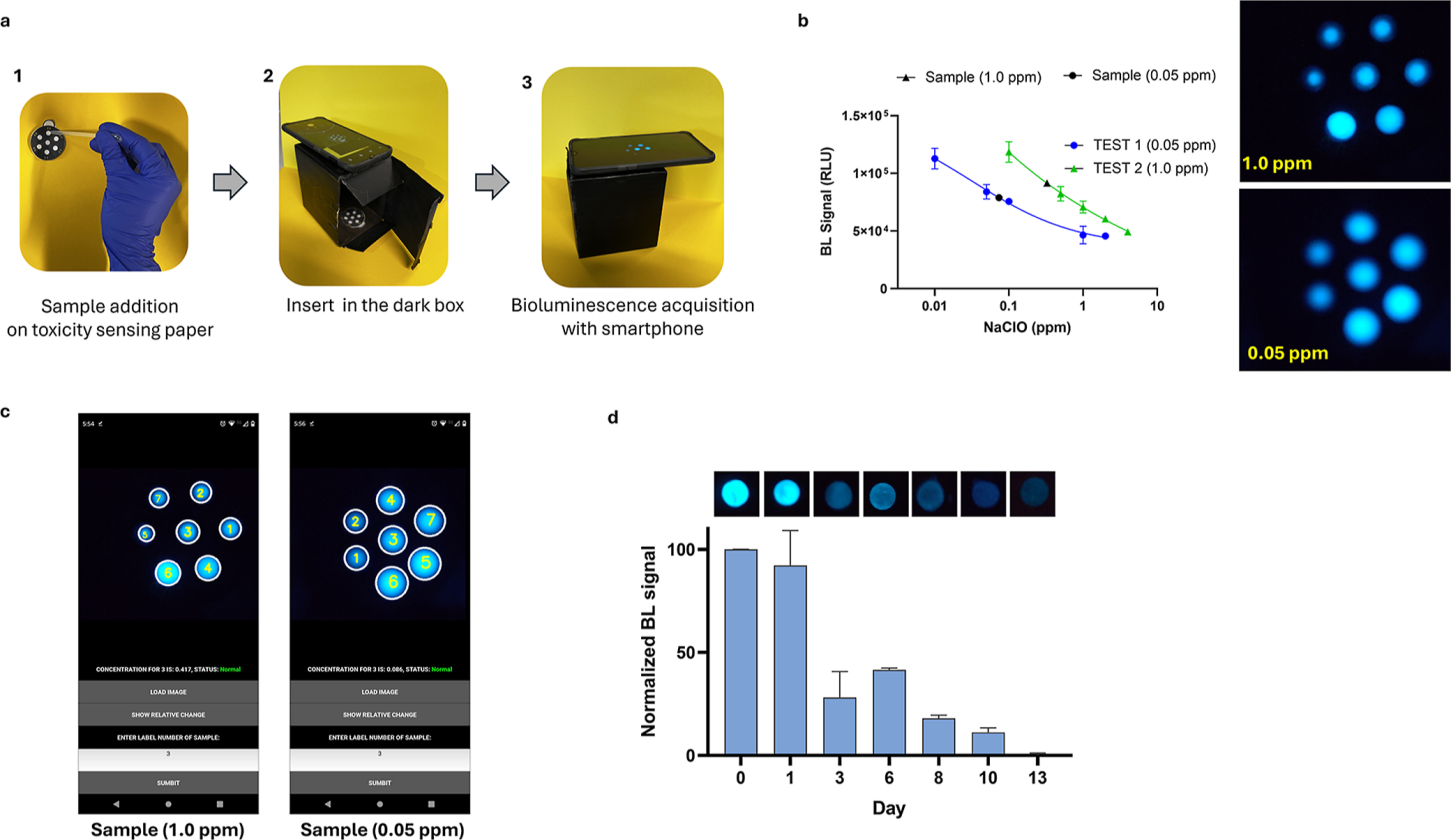
(a) Representation of the procedure for toxicity assessment with the sensing paper containing six external wells (diameter 0.5 mm) used for the calibration curve and one well for sample testing. (1) Deposition of 30 μL volume of standard solutions and sample into the paper-based sensor. (2) Setup of the signal acquisition using a dark box and a smartphone. (3) Picture of the sensor in the dark box. (b) Images of the toxicity sensing paper incubated with 0.05 and 1.0 ppm of NaClO for 1 min and acquired with the OnePlus 6T smartphone and calibration curves. (c) Read-out of the *Scentinel* app providing the “status” of the sample (normal) and interpolated concentration. (d) Stability of the sensing paper stored at +4 °C for 13 days. BL signals are acquired with OnePlus 6 (30 s at ISO 1600) and analyzed with ImageJ. BL images obtained on different days are normalized with data on day 0.

NaClO was used as a model analyte to test drinking water samples, which were spiked with three different concentrations (0.05, 1.0, and 4.0 ppm) and analyzed using the toxicity sensing paper after 1 min of incubation time at 25 °C. As shown in Figure 6b, the intensity of light emitted by the central well containing the sample is compared to the control (0 ppm) and the calibration solutions (0.1–4.0 ppm). For the sample with 0.05 ppm of NaClO, the calibrators range from 0.05 to 2.0 ppm. For the samples with 1.0 and 4.0 ppm of NaClO, the calibrators range from 0.5 to 4.0 ppm. Even a preliminary comparison achieved by the naked eye confirmed that all three samples showed intensities similar to those of the corresponding wells on the calibration curve. Figure 6c shows the corresponding values obtained with the *Scentinel* app and the graphs obtained with the standard ImageJ analysis and the values were interpolated using the NaClO calibration curve (Figures S13 and S14, Table S4).

### Stability and Reproducibility Studies

The stability and responsiveness of *A. fischeri* bacteria entrapped on paper with the agarose hydrogel and stored at 4 °C were evaluated for a two-week period. As shown in Figure 6d, although we observed a proportional decrease in BL intensities during the days, the toxicity sensing paper response was maintained within 13 days (about 48% of the initial response on day 6). At the same time, triplicate wells were incubated with 1 ppm of NaClO for 1 min, and BL intensities were acquired with the OnePlus 6 camera.

### Sustainability Assessment

Ensuring the sustainability of a biosensor is essential for reducing environmental impact in healthcare and environmental monitoring.^32^ The sustainability of the toxicity sensing paper was evaluated using the RGB 12 algorithm which incorporates 12 principles of white analytical chemistry (WAC).^33^ The developed toxicity sensor was compared to three previously reported laboratory-based standard methods, which utilized freshly prepared, liquid-dried, and freeze-dried *A. fischeri* bacteria for assessing the toxicity of water samples. Data shown in Supporting Information report the RGB scores of these methods as well as the score assessment criteria. In terms of sustainability (Green score), the smartphone-based toxicity sensor ranked first with a score of 89.2%, compared to 59.6% for the analogous assay performed with standard methods (ISO 11348). Standard methods and test kits involve the use of chemical reagents that require careful disposal to avoid secondary contaminations, plastic cuvettes, and microtiter plates. To reduce the environmental impact, our toxicity sensing paper was designed to be environmentally friendly thanks to the green properties of the paper, low sample volumes, and no additional steps of rehydration. Considering the feasibility, our method achieved a blue score of 100%, as it does not require benchtop or sophisticated instrumentation such as luminometers or strict cold chain (Tables S5, S6, and S7). Therefore, unlike the standard laboratory methods, the developed sensing paper allows for easy and rapid monitoring of water toxicity. It is highly suitable for integration with portable detectors for point-of-need analysis. Additionally, it offers the advantages of low cost and a low carbon footprint. The blue score was notably increased due to the reduced time and cost efficiencies of the developed system. The AI *Scentinel* App, compared to the ImageJ tool analysis, allowed a reduction in the total time of the analysis from about 1 h to 5 min avoiding the use of a laptop for data elaboration and software for graphical elaboration and statistical analysis.

## Conclusions

This work presents a sustainable, paper-based biosensor using bioluminescent marine bacteria for rapid water toxicity assessment. This is the first report in which bioluminescence detection has been combined with AI algorithms to avoid manual signal elaboration and to provide a truly user-friendly interface. A flower-like paper sensor configuration was designed to integrate an onboard calibration curve with a model toxic analyte, thus enabling the evaluation of toxicity of a water sample in terms of toxicity equivalents.

The analytical performance of the biosensor was assessed with harmful substances such as disinfectants, toxins, and heavy metals, showing the required sensitivity in tap and wastewater samples. While traditional lab methods remain standard, especially for assessing toxicological information, such as the effective concentration causing 50% luminescence inhibition, this portable system is ideal for quick screenings in diverse settings, especially in low-resource areas. The integrated Android app with AI-driven image analysis provides instant results, minimizing the need for technical skills and supporting citizen science. Future developments aim to enhance performance with broader data sets and expand testing to polluted sources, such as agricultural runoff. With its affordability (about €0.20 per cartridge), ease of use, and remote accessibility, the sensor supports better water quality monitoring and environmental health.

## Supplementary Material



## Notes

Experimental section (heat conduction calculation, *A. fischeri* entrapment optimization, recovery studies); S*centinel* app development and guide for users; and assessment of sustainability (red, green, and blue principles) (PDF)
