# Supplementary material for: An All-in-One Sustainable Smartphone Paper Biosensor for Water Toxicity Monitoring Combining Bioluminescence Detection with Artificial Intelligence
Source: Anal Chem. 2025 Aug 12;97(33):18092–100. doi: 10.1021/acs.analchem.5c02369 (PMC12392259; doi:10.1021/acs.analchem.5c02369)
Supplement: Supplementary file 1 [file ac5c02369_si_001.pdf]

Supplementary Materials

All-in-one sustainable smartphone paper biosensor for water toxicity monitoring combining bioluminescence detection with artificial intelligence

Faisal Nazir[†,‡], Denise Gregucci[†,‡], Maria Maddalena Calabretta[†], Caterina Cambrea[†], Peyman Vahidi[†], Stevo Lavrnić[¥], Attilio Toscano[¥], Elisa Michelini[†,x*]

[†]*Department of Chemistry "Giacomo Ciamician", University of Bologna, Via P. Gobetti 85, 40129, Bologna, Italy*

[¥] *Department of Agricultural and Food Sciences, University of Bologna, Viale Fanin 50, 40127, Bologna, Italy*

[x] *IRCCS Azienda Ospedaliero-Universitaria di Bologna, 40138 Bologna, Italy*

[‡]Faisal Nazir and Denise Gregucci contributed equally.

*Corresponding author:
Prof. Elisa Michelini
University of Bologna
Dept. of Chemistry "Giacomo Ciamician"
Via P. Gobetti 85, 40129 Bologna, Italy
elisa.michelini8@unibo.it

TABLE OF CONTENTS

EXPERIMENTAL SECTION 4

Heat conduction calculation 4

Figure S1: Bioluminescent signal of *A. fischeri* cells (a) with an OD600 value of 3.0 and 5.0, and (b) with OD600 of 5.0 entrapped in agarose 0.5%w/v, agarose 0.25%w/v + glycerol 15%w/v, and agarose 0.25%w/v + trehalose 10%w/v. 4

Figure S2: Normalized bioluminescent signal of *A. fischeri* cells entrapped in a) agarose 0.5%w/v and in b) agarose 0.25%w/v + trehalose 10%w/v hydrogel incubated with NaClO (concentration range 0-4 ppm) for 1 min at room temperature. 5

Figure S3: Bioluminescence emissions of *A. fischeri* entrapped in agarose 0.5%w/v incubated at room temperature for 1, 3, 5, 15, and 30 minutes with NaClO 0.0, 0.5, 2.0, and 4.0 ppm. Images were acquired with OnePlus 6T smartphone camera with ISO 1600 and 30 s of acquisition time. 5

Figure S4: Normalized bioluminescent signal of *A. fischeri* cells entrapped in a) agarose 0.5%w/v and in b) agarose 0.25%w/v + trehalose 10%w/v hydrogel incubated with increasing concentrations of 3,5-dichlorophenol for 1 min, 15 and 30 min. 6

Figure S5: Normalized bioluminescent signal of *A. fischeri* cells entrapped in a) agarose 0.5%w/v and in b) agarose 0.25%w/v + trehalose 10%w/v hydrogel incubated with increasing concentrations of Microcystin-LR for 1 min, 15 and 30 min. 6

Figure S6: Normalized bioluminescent signal of *A. fischeri* cells entrapped in a) agarose 0.5%w/v and in b) agarose 0.25%w/v + trehalose 10%w/v hydrogel incubated with increasing concentrations of lead nitrate for 1 min, 15 and 30 min. 7

Figure S7: Normalized bioluminescent signal of *A. fischeri* after addition of 30 and 50 µL of NaClO solutions and incubation time of 1 min, and images acquired with the OnePlus 6T smartphone camera (ISO 1600, integration time of 30 sec). 7

Figure S8: Normalized bioluminescent signal of *A. fischeri* after addition of 30 and 50 µL of Microcystin-LR solutions and incubation time of 15 min, and images acquired with the OnePlus 6T smartphone camera (ISO 1600, integration time of 30 sec). 8

Figure S9: Normalized bioluminescent signal of *A. fischeri* after addition of 30 and 50 µL of 3,5-dichlorophenol solutions and incubation time of 15 min, and images acquired with the OnePlus 6T smartphone camera (ISO 1600, integration time of 30 sec). 8

Figure S10: Normalized bioluminescent signal of *A. fischeri* after addition of 30 and 50 µL of 3,5-lead nitrate solutions and incubation time of 15 min, and images acquired with the OnePlus 6T smartphone camera (ISO 1600, integration time of 30 sec). 9

Figure S11: NaClO dose-response curves in (a) drinking water and (b) in wastewaters obtained with the paper-based biosensor (1-min incubation time). BL signals were acquired with OnePlus6 smartphone camera (30-sec integration time, ISO 1600) and quantified by ImageJ software (colored) and the AI assisted application (black). 9

Table S1: Recovery studies in water samples (tapwater and wastewater samples) spiked with NaClO 1ppm, MC-LR8 ppb, DCP 3.5 ppm and Pb 35 ppb. Recovery was calculated as follows: Recovery % =[recovered (measured spiked sample-baseline)/ concentration added] x 100 10

Table S2: Limits of detections for NaClO obtained by analysing the pictures of different smartphone models either with ImageJ and the *Scentinel app*. The procedure is described in Materials and Methods section. 11

Figure S12: The paper sensor printed with an office wax printer onto chromatography paper. The paper biosensor includes 6 wells for the calibration curve and a central well for the sample. 11

Scentinel App development and guide for users 11

Pre-processing of Image 12

Image Segmentation and Extraction of Properties 12

Step by step guide for Scentinel app use 13

Comparison between Manual and AI based analysis 14

Figure S13. Regions of interest (ROIs) of the toxicity paper sensor with NaClO concentrations in ppm, measured with AI based analysis (a) and with manual segmentation with Image J software (b). The lower panel shows the calibration curves obtained with either Image or the Scentinel App. 15

Disposable flower-like paper toxicity biosensor evaluation 15

Table S3: Comparison of manually interpolated values and the application interpolated values along with the standard deviation. 16

Practical Application of Biosensor (stability, reproducibility, technological improvement AI) 16

Technological Improvement by AI-based detection 16

Figure S14. Right panel shows the Scentinel application operational process in smartphone (a) Flowchart illustrating the operational process of the Scentinel application, detailing the steps from application loading and image selection to the final statistical analysis and results presentation, including server interaction and user input. (b) operational process of manual interpolation of results requiring different software along with smartphone 17

ASSESSMENT OF SUSTAINABILITY 17

Red principles 18

Table S4: Criteria assignment of red principles scores according to Nowak et al. for the efficiency assessment of bioluminescent cell-based methods relying on paper-based or standard approaches. For comparison we are using standard ISO methods, and we compared only the precision of the methods. Other analytical parameters are not provided by ISO. 18

Green principles 18

Table S5: Criteria assignment of green principles scores according to Nowak et al. for the sustainability assessment of cell-based methods. Pictograms of the 'Globally Harmonized System of Classification and Labelling of Chemicals", for energy consumption kWh are considered. 19

Blue principles 20

Table S6: Criteria assignment of blue principles scores according to Nowak et al. for the economic assessment of bioluminescent cell-based methods relying on paper-based or standard approaches. 21

REFERENCES 23

EXPERIMENTAL SECTION

Heat conduction calculation

Heat conduction calculation of the bacterial suspension - agarose was investigated to avoid the effect of high temperature of agarose hydrogel on the *Aliivibrio fischeri* vitality. By applying Richmann's law[1] to solutions of agarose mixed with LB containing bacteria the temperature change was approximated using equation:

$$T_{\mathrm{f}} = \frac{V_1 \cdot T_1 + V_2 \cdot T_2}{V_1 + V_2} \qquad (1)$$

where T_f is final temperature of solution, C_1 and C_2 are specific heat capacities of solutions, V_1 and V_2 are volumes of solutions and $T_1$and T_2 are their respective temperatures at the time of mixing. The agarose's specific heat capacity[2] is 2.85 J/g°C and volume and temperature are 80 µL and 60°C respectively, while the specific heat capacity of LB is considered 4.2 J/g°C, higher near to water. The volume of LB with bacteria is 420 µL, with the temperature of 19°C. The calculated rise in temperature was of about 30°C, not enough to destroy bacterial cells.

Figure S1: Bioluminescent signal of *A. fischeri* cells (a) with an OD600 value of 3.0 and 5.0, and (b) with OD600 of 5.0 entrapped in agarose 0.5%w/v, agarose 0.25%w/v + glycerol 15%w/v, and agarose 0.25%w/v + trehalose 10%w/v.

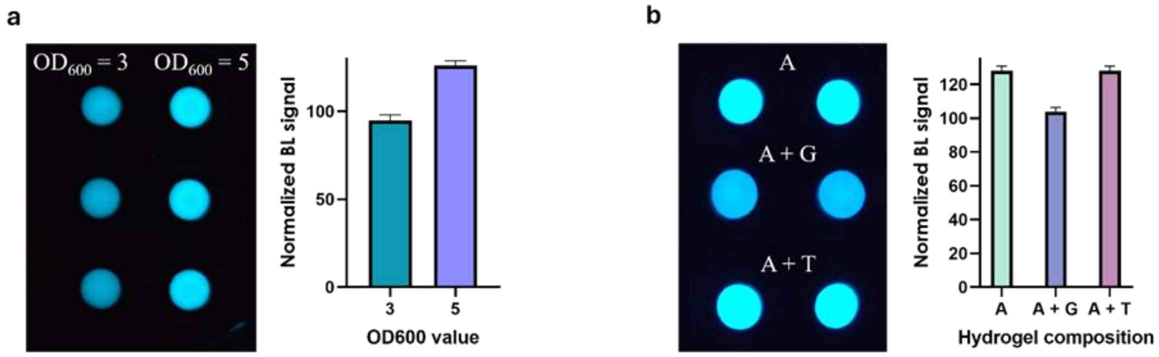

Figure S2: Normalized bioluminescent signal of *A. fischeri* cells entrapped in a) agarose 0.5%w/v and in b) agarose 0.25%w/v + trehalose 10%w/v hydrogel incubated with NaClO (concentration range 0-4 ppm) for 1 min at room temperature.

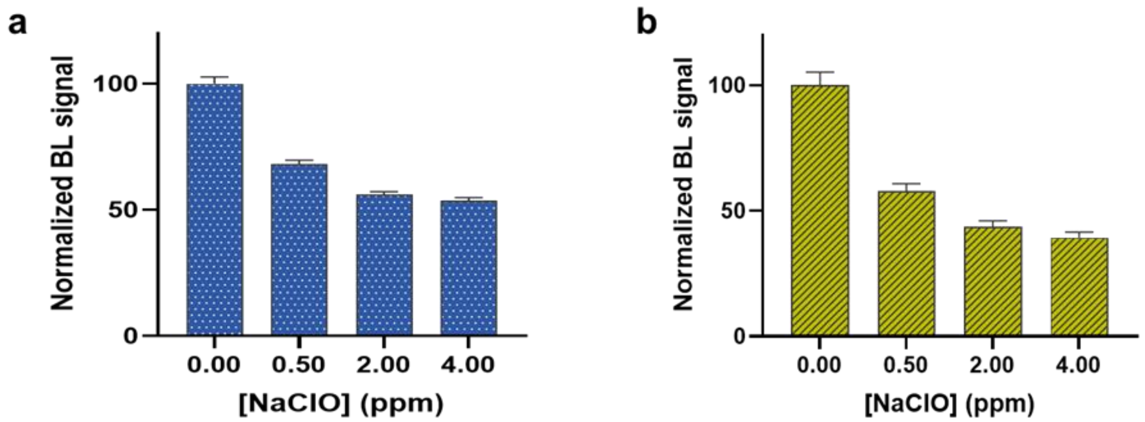

Figure S3: Bioluminescence emissions of *A. fischeri* entrapped in agarose 0.5%w/v incubated at room temperature for 1, 3, 5, 15, and 30 minutes with NaClO 0.0, 0.5, 2.0, and 4.0 ppm. Images were acquired with OnePlus 6T smartphone camera with ISO 1600 and 30 s of acquisition time.

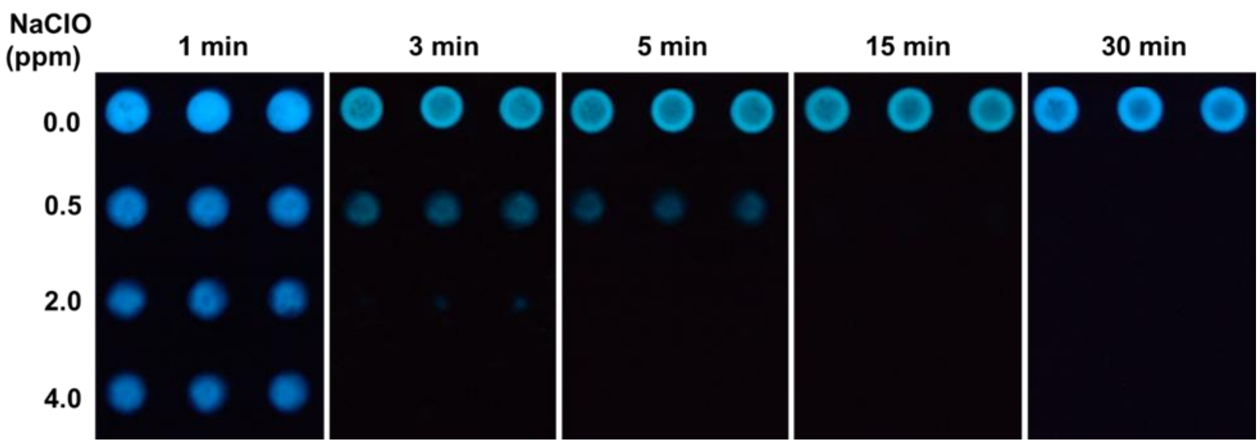

Figure S4: Normalized bioluminescent signal of *A. fischeri* cells entrapped in a) agarose 0.5%w/v and in b) agarose 0.25%w/v + trehalose 10%w/v hydrogel incubated with increasing concentrations of 3,5-dichlorophenol for 1 min, 15 and 30 min.

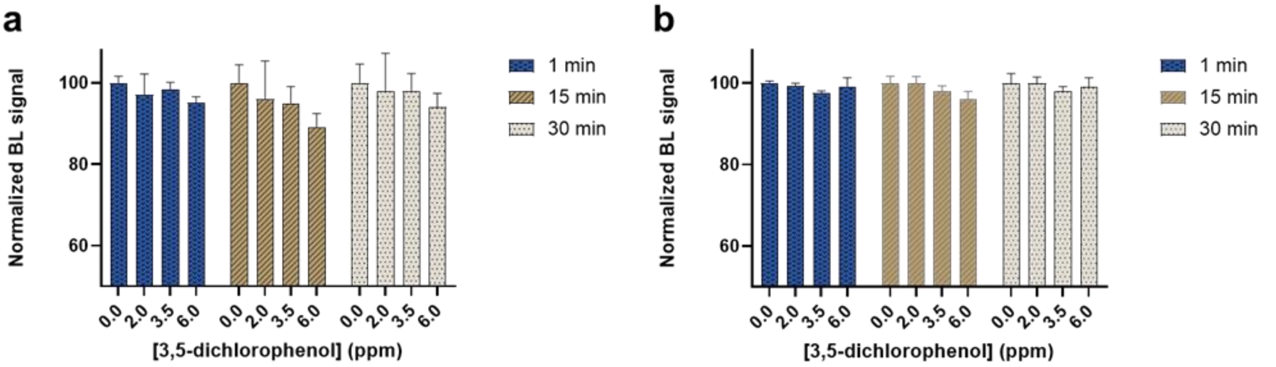

Figure S5: Normalized bioluminescent signal of *A. fischeri* cells entrapped in a) agarose 0.5%w/v and in b) agarose 0.25%w/v + trehalose 10%w/v hydrogel incubated with increasing concentrations of Microcystin-LR for 1 min, 15 and 30 min.

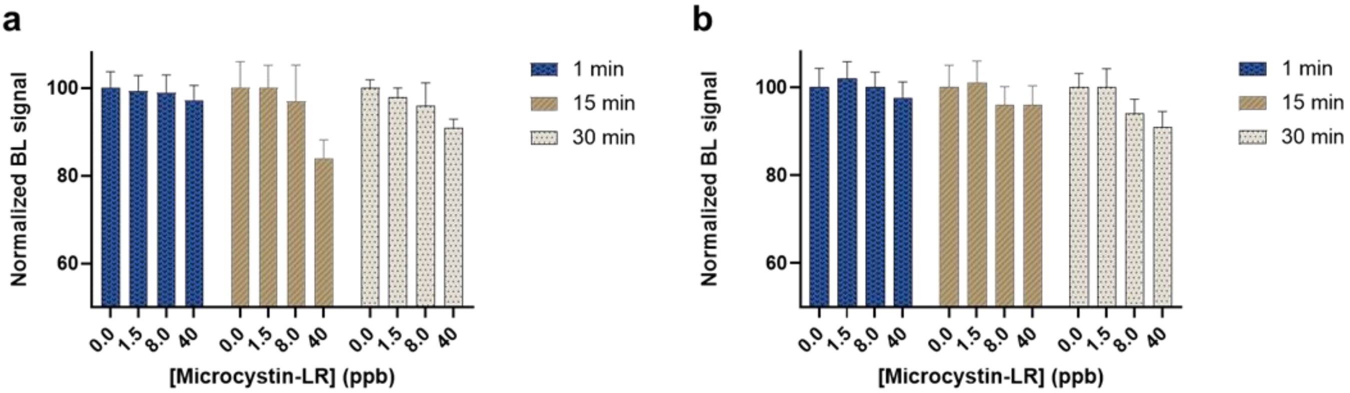

Figure S6: Normalized bioluminescent signal of *A. fischeri* cells entrapped in a) agarose 0.5%w/v and in b) agarose 0.25%w/v + trehalose 10%w/v hydrogel incubated with increasing concentrations of lead nitrate for 1 min, 15 and 30 min.

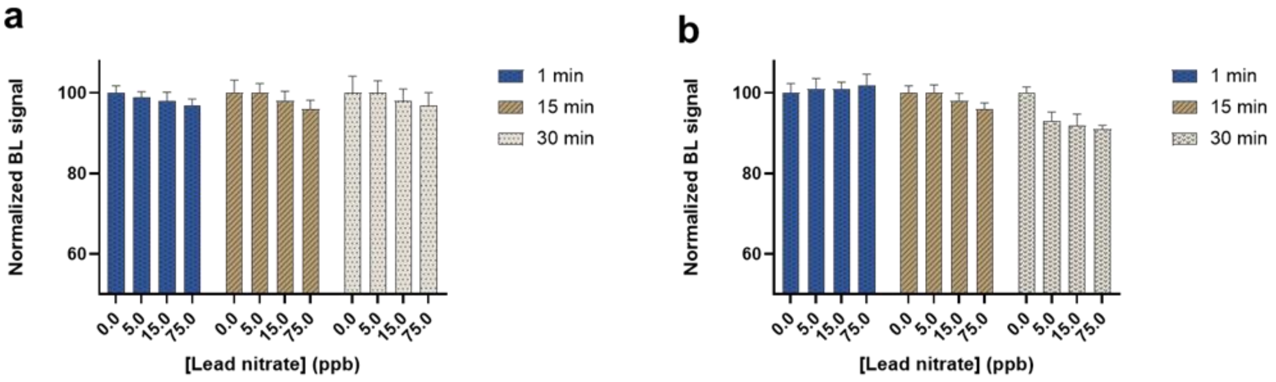

Figure S7: Normalized bioluminescent signal of *A. fischeri* after addition of 30 and 50 µL of NaClO solutions and incubation time of 1 min, and images acquired with the OnePlus 6T smartphone camera (ISO 1600, integration time of 30 sec).

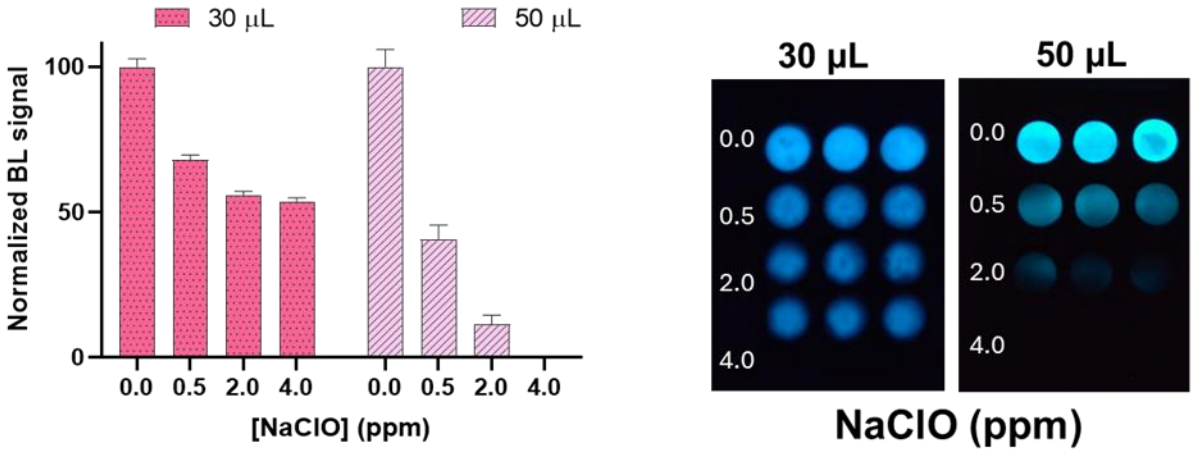

Figure S8: Normalized bioluminescent signal of *A. fischeri* after addition of 30 and 50 µL of Microcystin-LR solutions and incubation time of 15 min, and images acquired with the OnePlus 6T smartphone camera (ISO 1600, integration time of 30 sec).

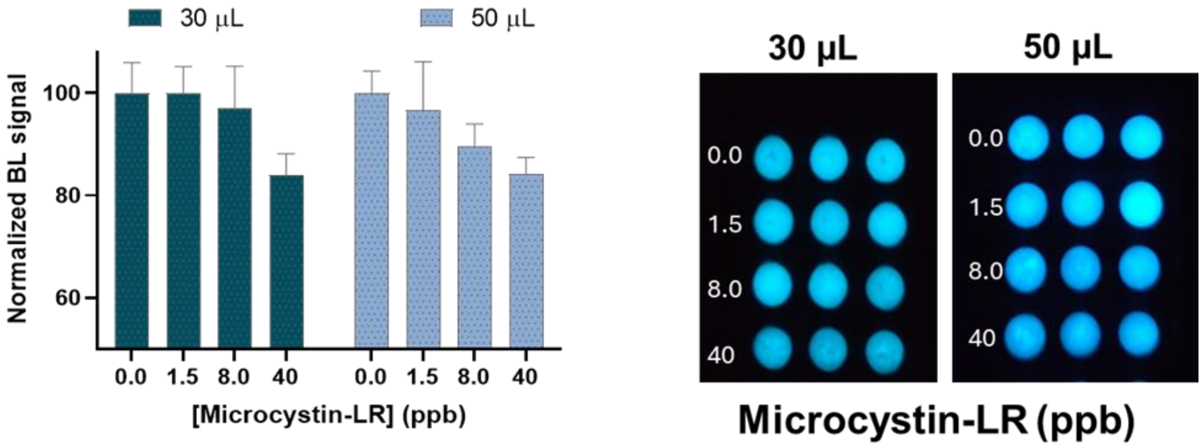

Figure S9: Normalized bioluminescent signal of *A. fischeri* after addition of 30 and 50 µL of 3,5-dichlorophenol solutions and incubation time of 15 min, and images acquired with the OnePlus 6T smartphone camera (ISO 1600, integration time of 30 sec).

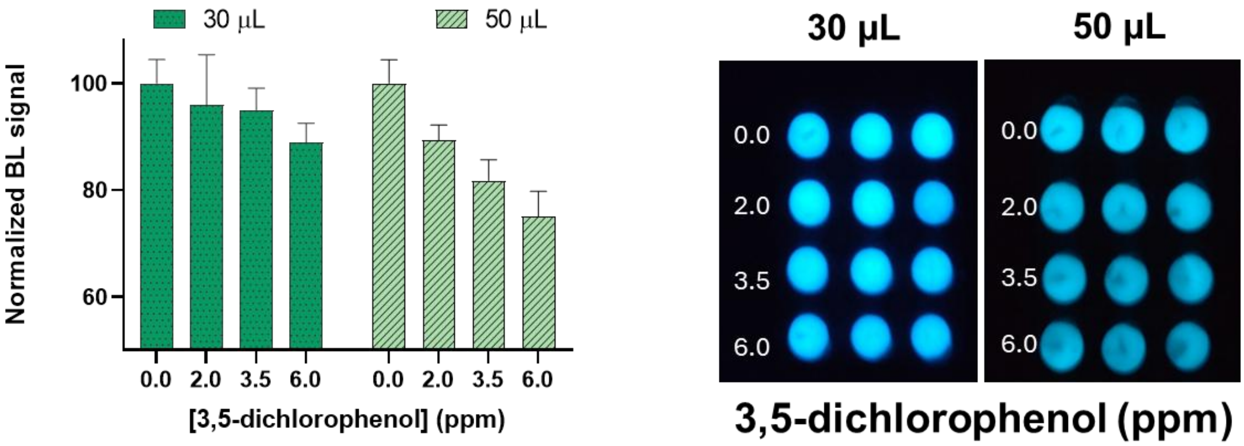

Figure S10: Normalized bioluminescent signal of *A. fischeri* after addition of 30 and 50 µL of 3,5-lead nitrate solutions and incubation time of 15 min, and images acquired with the OnePlus 6T smartphone camera (ISO 1600, integration time of 30 sec).

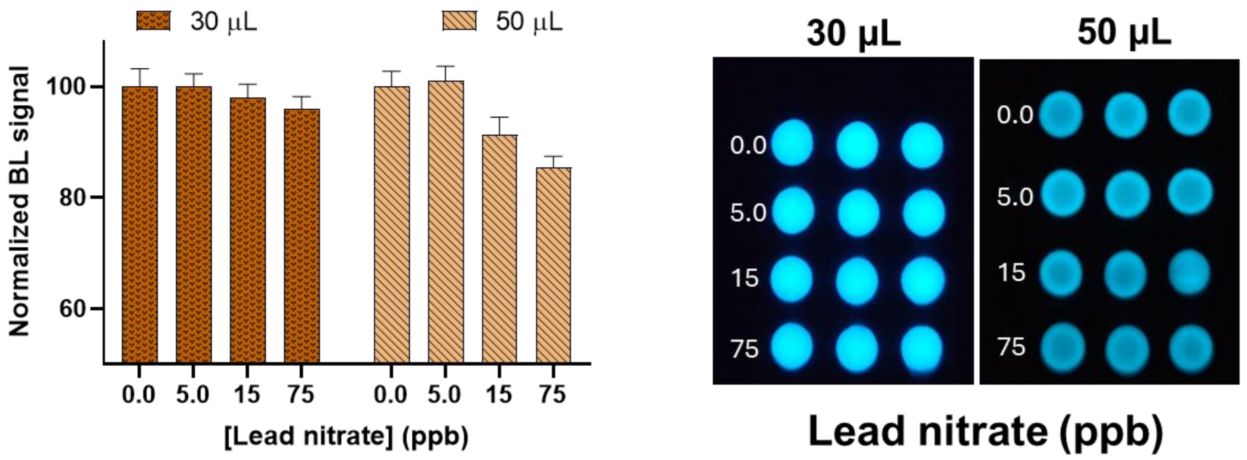

Figure S11: .NaClO dose-response curves in (a) drinking water and (b) in wastewaters obtained with the paper-based biosensor (1-min incubation time). BL signals were acquired with OnePlus6 smartphone camera (30-sec integration time, ISO 1600) and quantified by ImageJ software (colored) and the AI assisted application (black).

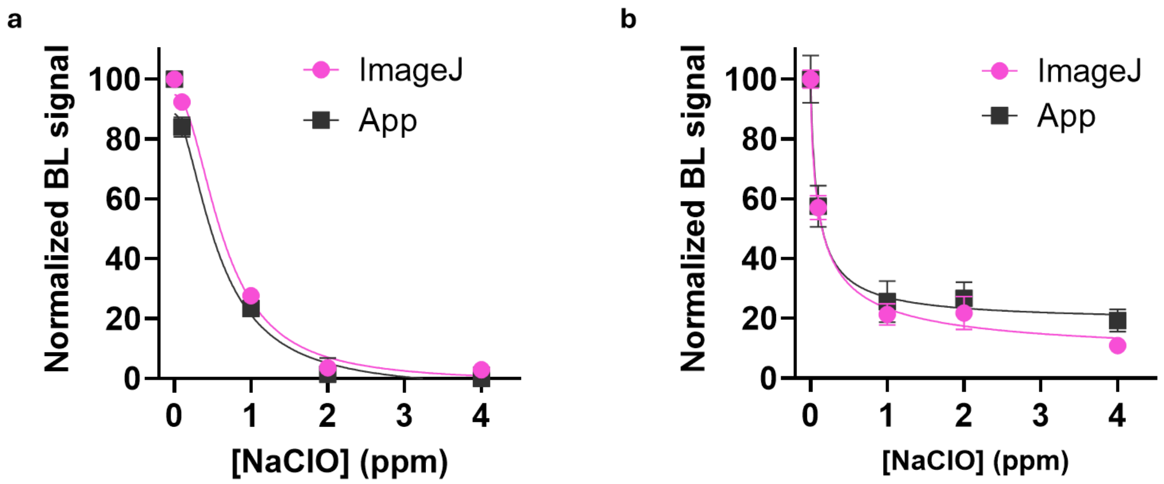

Table S1: Recovery studies in water samples (tapwater and wastewater samples) spiked with NaClO 1 ppm, MC-LR 8 ppb, DCP 3.5 ppm and Pb 35 ppb. Recovery was calculated as follows: Recovery % =[recovered (measured spiked sample-baseline)/ concentration added] x 100

Recovery % for NaClO 1 ppm

	**ImageJ**	**Scentinel**
Tap water	77.7	73.4
Wastewaters	59.6	80.3

Recovery % for MC-LR 8 ppb

	**ImageJ**	**Scentinel**
Tap water	83.1	116.5
Wastewaters	91.7	106

Recovery % for DCP 3.5 ppm

	ImageJ	Scentinel
Tap water	97.7	90.9
Wastewaters	97.4	95.7

Recovery % for Pb 35 ppb

	ImageJ	Scentinel
Tap water	110	105
Wastewaters	103	104

Table S2: Recovery studies in tap water samples spiked with different concentrations of NaClO , MC-LR, DCP and Pb. Recovery was calculated as follows:
Recovery % =[recovered (measured spiked sample-baseline)/ concentration added] x 100

NaClO in tap water

[NaClO] ppm	**ImageJ**	**Scentinel**
0	100	100
0.1	93.5	79.6
0.5	98.4	87.2
1.0	77.7	73.4
2.0	29.0	12.8
4.0	20.6	n.d.

MC-LR in tap water

[MC-LR] ppb	**ImageJ**	**Scentinel**
0	100	100
1.5	94.8	117
5.0	85.6	115
8.0	83.1	117
18	87.3	119
40	105	142

DCP in tap water

[DCP] ppm	**ImageJ**	**Scentinel**
0	100	100
1.0	103	98.5
2.0	98.0	91.7
3.5	97.7	90.9
5.0	98.7	107
6.0	112	117

Pb in tap water

[Pb] ppb	**ImageJ**	**Scentinel**
0	100	100
5.00	106	98.3
15.0	111	102
35.0	110	105
75.0	120	116
100	120	120

Table S3: Limits of detection for NaClO obtained by analysing the pictures of different smartphone models either with ImageJ and the *Scentinel app*. The procedure is described in Materials and Methods section.

Smartphone model	LOD (ppm) obtained with ImageJ-based analysis	LOD (ppm) obtained with the *Scentinel app*
OnePlus6	0.10	0.21
Motorola edge 40 neo	0.06	0.06
SamsungS20	0.06	0.06
HuaweiP10	0.29	0.31
iPhone12mini	0.40	0.46
iPhone13mini	0.43	0.59

Figure S12: The paper sensor printed with an office wax printer onto chromatography paper. The paper biosensor includes 6 wells for the calibration curve and a central well for the sample.

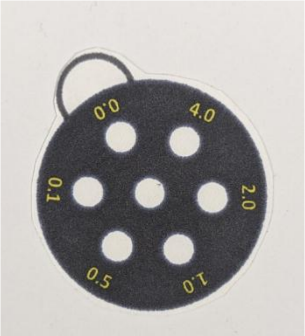

Scentinel App development and guide for users

The Scentinel application provides a simple graphical user interface (GUI) (basic flow of work is described in Figure S14, written using Kivy[3] framework of python, to upload the captured images from the smartphone gallery. The image processing functionality is deployed on Microsoft Azure[4], utilizing Flask's API[5], a server-side web framework for managing HTTP requests and responses. The quantification of sample concentration is performed on the smartphone.

In the first phase, a Python[6] program was developed within an integrated development environment (IDE), focusing on code validation, statistical analysis through curve fitting, and adjustments of AI parameters. In the second phase, a program to application transition was performed. The probability parameter and non-maximum suppression (NMS) for the AI model were fine-tuned to 0.67 and 0.01 respectively to increase confidence level for prediction of bioluminescence signal and filtering low level of signals.

Pre-processing of Image

In this step, the input image is pre-processed for faster segmentation aiming to utilize fewer computing resources such as bandwidth and storage, while aligning with the AI model. First, the image decimation is performed preserving the aspect ratio, followed by converting to grayscale by applying Eq. (2) to each pixel[7]. In the equation R, G and B are integers representing red (R), green (G) and blue (B) with values from 0 to 255. Finally, the pixel values of the image are normalized before being fed to the AI model.

$$Y = 0.299\ R + 0.587\ G + 0.114\ B \qquad (2)$$

Image Segmentation and Extraction of Properties

In the application, StarDist[8] library was utilized, commonly applied for cell detection and segmentation in microscopy images. StarDist predicts star-convex polygons rather than traditional axis-aligned bounding boxes by regressing radial distances from each pixel to object boundaries. Object probabilities are derived from the Euclidean distance to background pixels, with non-maximum suppression (NMS) refining the final object instances[9]. The pre-trained model 'Versatile (fluorescent nuclei)' of StarDist was employed to segment each well, representing bioluminescence signal, on the sensor image. Subsequently, measuring properties (area, bounding box) of detected well was performed by scikit-image algorithms[10]. The bioluminescence value of each well was calculated by dividing its total pixel values to the total number of pixels of control. Then OpenCV[11] library was used to draw circles around segmented wells and to label them with numbers. Coding files are freely

available in GITHUB repository (https://github.com/faisalnazir1213/ScentinelRegression curve fitting and interpolation of the sample value

In this phase, based on user input, the bioluminescence signal value assigned to the sample well (unknown) is extracted, separating it from the known wells (calibration solutions). The application then prompts the user to input the concentration values of the calibration solutions. This is necessary to provide actual values to the corresponding captured signal values in order to perform statistical analysis. If the user submits without entering values, the relative toxicity is calculated using the default NaOCl calibration values (0.0 ppm, 0.1 ppm, 0.5 ppm, 1.0 ppm, 2.0 ppm, 4.0 ppm). Alternatively, the user can manually enter the concentration values.

Then a 4-parametric regression model is implemented using SciPy library on calibration solution correlating each concentration to its corresponding signal value using Eq. (3). Then, the app computes the final measurement by interpolating the sample concentration with respect to the calibration curve using Eq. (4). Where x is the independent variable (concentration values of calibration solutions), y is the dependent variable (bioluminescence values of calibration solutions), A is the minimum value that can be obtained, B is the Hill's slope of the curve, C is the point of inflection, and D is the maximum value that can be obtained.

$$y = \frac{A - D}{1 + \left(\frac{x}{C}\right)^{B}} + D \qquad (3)$$

$$x = C\left(\frac{A - D}{y - D} - 1\right)^{\frac{1}{B}} \qquad (4)$$

Step by step guide for Scentinel app use

Step 1: Open the App and Load Your Image

A. Start by launching the application on your android device.

B. Click on the 'LOAD IMAGE' button here you can upload/select an image of biosensor. Choose the image you wish to analyze.

C. The processed image will be displayed assigning all wells with numbers (the sample well number will be used for further analysis)

Step 2: View Relative Changes (Optional)

D. Once the image is loaded, locate and click the ‘SHOW RELATIVE CHANGE’ button. This button is found under the ‘LOAD IMAGE’ button.

E. After clicking, the app will display the relative changes/signal values of each well on the new prompt. These values represent the intensities of wells.

Step 3: Enter Sample Well Number

F. To proceed with the analysis, you need to specify the sample well number. This is done by entering the number into a designated input box under text ‘ENTER LABEL NUMBER OF SAMPLE’.

G. Ensure you enter the correct number corresponding to your sample (central well). This step is crucial for the app to associate the correct data with your sample.

H. After entering the sample well number, click the ‘SUBMIT’ button to finalize this step.

Step 4: Enter Calibration Solution Concentrations

I. In a new prompt of the app, you will be asked to enter the calibration solution concentrations. These concentrations must be numbers (e.g., 0, 1, 2, 3, 4...).

J. Input the calibration solution concentrations in the increasing order (4, 3,2,1,0…)

K. After entering all the required concentrations, press the ‘SUBMIT’ button. The app will then process this data to estimate the tentative concentration of the sample.

Step 5: Review Tentative Concentration

L. Once the app has processed the calibration solution concentrations, it will display the tentative concentration of the sample on the screen under the labelled image. This value is an estimate based on the data entered and the calibration solutions used.

Comparison between Manual and AI based analysis

Before developing the android based Smartphone application, a comparative analysis was conducted between the results of AI-based analysis procedures (Fig.S13a) and with manual ImageJ software (Fig. S13b). The paper sensor, printed with 4 x 4 arrays of wells, immobilized with *Aliivibrio fischeri*

was prepared and concentrations of NaClO (0, 0.01, 0.05, 0.1, 0.5, 1, 2, 4 ppm) were introduced to it. After the proposed duration images were acquired under dark box and analyzed to obtain values of bioluminescence signal. The curve was fitted (Fig. S13c) on the values using GraphPad Prism 8 to determine the limit of detections (LOD) those were 0.039 ppm for manual and 0.044 ppm for AI-based approach, validating the model's suitability for detection and quantification. However, it is noteworthy to mention that due to the absence of zone boundaries in higher concentrations 4ppm (wells below 13 and 14) due to no bioluminescence signal as displayed in the last row of Fig. S13a. the AI model could not segment and detect anything.

Figure S13. Regions of interest (ROIs) of the toxicity paper sensor with NaClO concentrations in ppm, measured with AI based analysis (a) and with manual segmentation with Image J software (b). The lower panel shows the calibration curves obtained with either Image or the Scentinel App (c).

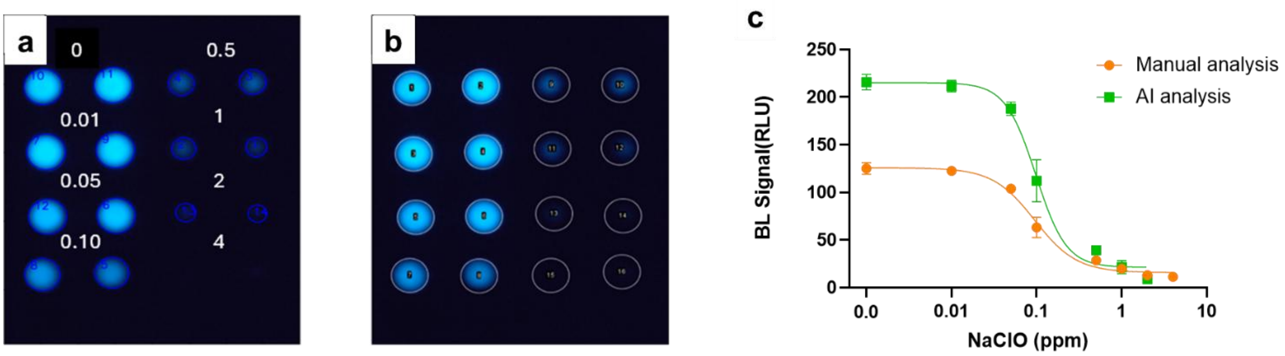

Disposable flower-like paper toxicity biosensor evaluation

To enable users to quantify samples on-site using the *Scentinel Application*, a disposable flower-like paper toxicity biosensor was developed, with a calibration curve established around the central sample well (Figure 6c). Different concentrations of NaClO (0.05, 1.0 ppm, and 4.0 ppm) were tested in the central well, the interpolated values from both methods, ImageJ and AI-based, were compared as shown in Table S4. The introduction of toxicity curve provides the solution to quantify relative toxicity according to calibration solutions of known concentrations. Both the *Scentinel application* and manual approach (computer-based opensource software ImageJ) produced similar results (Table

S4), unlike manual approach *Scentinel application* requires no effort from the user side except image acquisition.

Table S4: Comparison of manually interpolated values and the application interpolated values along with the standard deviation.

Manual Interpolation (ppm)	App-based Interpolation (ppm)	Standard Deviation(σ)
0.079	0.081	0.001
0.35	0.40	0.02
1.47	1.41	0.03

Practical Application of Biosensor (stability, reproducibility, technological improvement AI)

Technological Improvement by AI-based detection

Manual image processing and quantitative analysis require technical expertise, time, and access to hardware (e.g., personal computers) and software (e.g., image processing, statistical tools, spreadsheets), making participation in citizen science challenging. In contrast, our Android-based *Scentinel* application automates all steps previously performed manually, streamlining image analysis and quantification, and eliminating the need for bulky hardware, software and manual effort. The blue principles of the RGB 12 algorithm[12] discussed in a later section, further support the effectiveness of this smartphone-based approach, streamlining the process and enhancing accessibility for users without technical expertise.

Figure S14. Right panel shows the Scentinel application operational process in smartphone (a) Flowchart illustrating the operational process of the Scentinel application, detailing the steps from application loading and image selection to the final statistical analysis and results presentation, including server interaction and user input. (b) operational process of manual interpolation of results requiring different software along with smartphone

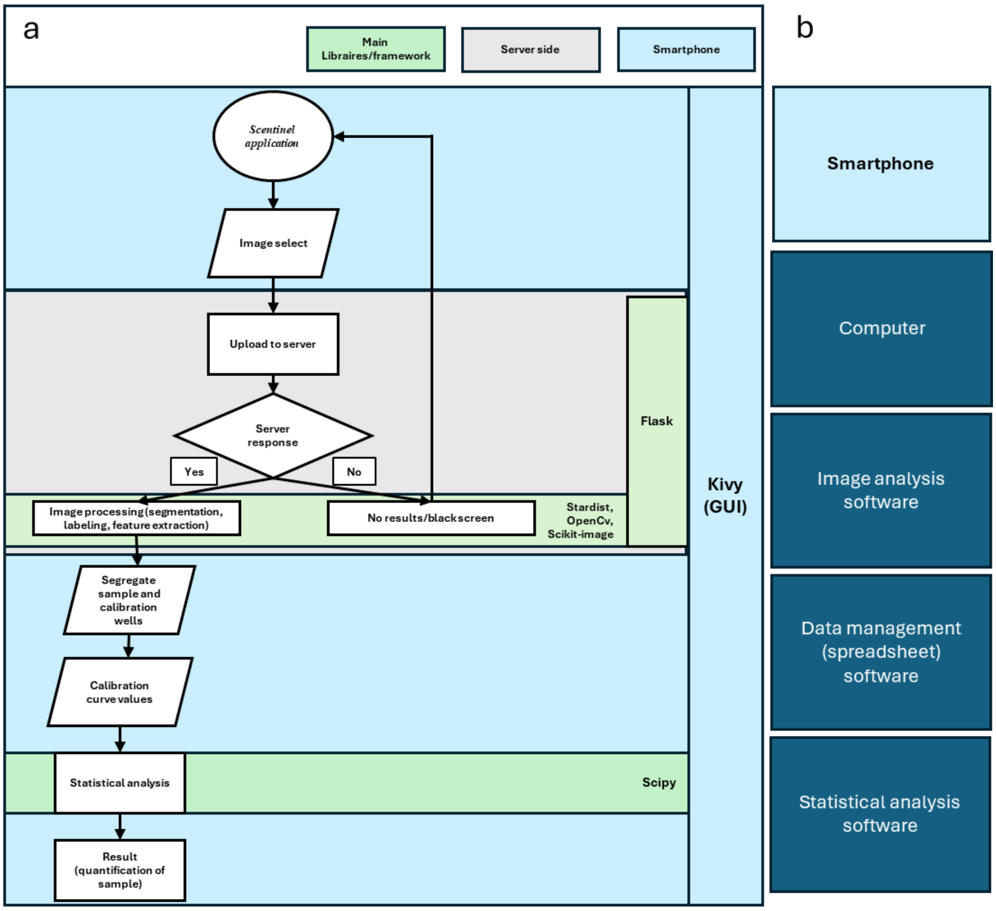

ASSESSMENT OF SUSTAINABILITY

The RGB algorithm includes three components: red for analytical efficiency, green for adherence to green analytical chemistry (GAC) principles, and blue for practical/economic efficiency and productivity, and is used for the assessment of the “whiteness” of the method. After evaluation, the method is assigned a color based on the proportions of each primary color. An optimal, comprehensive, and consistent analytical procedure is represented by a white method, indicating high saturation for each primary color. Conversely, a darker final color stands for a lower sustainability of the method.

Red principles

R1: Scope of application. Any given analytical method should be capable of being applied to a multitude of different situations and samples, as well as being able to determine a wide range of different analytes. It is crucial that it can be applied over a vast range of concentrations, and that it be compatible with a diverse range of sample types. Additionally, the method must demonstrate resilience to potential interferences.

R3: Precision. Precision is a fundamental aspect of analytical methods, and it is essential that the precision of these methods be characterized by the best possible repeatability and reproducibility of the results.

Table S5: Criteria assignment of red principles scores according to Nowak et al. for the efficiency assessment of bioluminescent cell-based methods relying on paper-based or standard approaches. For comparison we are using standard ISO methods, and we compared only the precision of the methods. Other analytical parameters are not provided by ISO.

Score	R1. Scope of Application	R3. Precision RSD (Reproducibility)
**100**	0 < pictograms ≤ 1	0 < % ≤ 5
**100 -75**	1 < pictograms ≤ 2	5 < % ≤ 10
**75 - 50**	2 < pictograms ≤ 5	10 < % ≤ 20
**50 - 25**	5 < pictograms < 10	20 < % ≤ 40
**25 - 0**	≥ 10 pictograms	≥ 40

Green principles

G1: Toxicity of reagents. It is of utmost importance that reagents exhibit minimal toxicity. When feasible, it is advisable to select biodegradable, renewable, or naturally occurring reagents and materials. In order to assess the safety of the reagents, the safety data sheets were evaluated, taking into account the quantity of pictograms for each reagent utilized in the cell-based method.

G2: Amount of reagents and waste. It is recommended that the use of reduced volumes be employed in order to minimize the waste.

G3. Energy and other media. In the development of an analytical method, it is essential to consider the lowest possible electricity consumption. Therefore, our assessment focused on the energy consumption required for the maintenance of cell cultures (incubators, safety cabinets, optical density meters, refrigerators, centrifuges, etc.) and for light detection (the use of benchtop luminometers, spectrophotometers, smartphones, and other portable detectors) without considering the storage conditions.

G4. Direct impacts. It is important to make sure that the deployment of an analytical method does not affect humans or animals. In this case, we focused on the impact on humans and the environment related to the reagents used for the cell-based assays.

Table S6: Criteria assignment of green principles scores according to Nowak et al. for the sustainability assessment of cell-based methods. Pictograms of the "Globally Harmonized System of Classification and Labelling of Chemicals", for energy consumption kWh are considered.

Score	G1. Toxicity of reagents	G2. Amount of reagents and waste	G3. Energy consumption	G4. Direct impact
**100**	0 < pictograms ≤ 1	0 < ml ≤ 0.5	Exceptionally low or non-consuming	No hazardous activity
**100 -75**	1 < pictograms ≤ 2	0.5 < ml ≤ 1	< 0.1 kWh	Exceptionally low hazardous activity
**75 - 50**	2 < pictograms ≤ 5	1 < ml ≤ 2.5	0.1 - 1.5 kWh	low hazardous activities
**50 - 25**	5 < pictograms < 10	2.5 < ml ≤ 5	> 1.5 kWh	medium hazardous activities
**25 - 0**	≥ 10 pictograms	> 5 ml	+ tools > 1.5 kWh	Dangerous activities

Blue principles

B1. Cost-efficiency. It is important to minimize the total cost as much as possible. In this context, the cost of all necessary reagents, materials, equipment, and personnel was taken into consideration in order to calculate the total analysis cost.

B2. Time-efficiency. A reduction in analysis time is a desirable outcome, encompassing method development and all steps of the workflow. In this context, the time required for preparation of the experimental set-up and incubation time with the sample were considered.

B3. Requirements. It is recommended to minimize the use of equipment, sample quantity, and skilled personnel. In this context, the necessity for incubators, luminometers, spectrophotometers, image analysis software, and statistical analysis software for the assay was considered.

B4. Operational simplicity. In the optimal scenario, an analytical method should be fully automated, employ artificial intelligence techniques, and should be miniaturized and portable, thus enabling on-site measurements.

Table S7: Criteria assignment of blue principles scores according to Nowak et al. for the economic assessment of bioluminescent cell-based methods relying on paper-based or standard approaches.

Score	B1. Cost Efficiency	B2. Time efficiency	B3. Requirements		B4. Operational Simplicity		
			B3.1 Sample consumption	B3.2 Other needs*	B4.1 Portability	B4.2 Integration automation	B4.3 Miniaturization
**100**	Very low-cost	1 < min ≤ 30	0 < ml ≤ 0.1	Simple Accessories & tools (mobile, pipette, dark box, etc.)	Online analysis	High automation or simple, online data	Handheld
**100 - 75**	Low-cost	30 < min ≤ 60	0.1 < ml ≤ 1	Simple Equipment (glassware, dark box, basic equipment for manual preparation, mobile/camera/ccd, image processing, statistical software)	At line analysis, camp laboratory	High automation or simple	Portable
**75 - 50**	Medium cost	1 < hrs. ≤ 2	1 < ml ≤ 2.5	Medium complexity instrumentation (spectrophotometer), statistical	In lab analysis	Partially automation	Transportable

				analysis, equipment for basic preparation			
**50 - 25**	Expensive	2 < hrs. ≤ 4	2.5 < ml ≤ 5	Complex instrumentation, equipment for preparation (Luminometer, statistical analysis, Incubator, Biological safety cabinet, Refrigerator)	In lab analysis with sample storing, batch working, results obtained the same day of analysis	No automation	Bench
**25 - 0**	Extremely expensive	≥ 1 Day	> 5 ml	High complexity instrumentation (luminometer, cell culturing equipment (centrifugation, storage, etc.), optical density meter, Statistical analysis software, other complex equipment	In lab analysis with sample storing, planned batch working, results obtain, days after the analysis	No automation	Complex facilities

REFERENCES

(1) Heilbron, J. L. *Elements of Early Modern Physics*; University of California Press, 1982.

(2) Myers, E.; Piazza, M.; Owkes, M.; June, R. K. Heat Conduction Simulation of Chondrocyte-Embedded Agarose Gels Suggests Negligible Impact of Viscoelastic Dissipation on Temperature Change. *J Biomech* **2024**, *176*, 112307.

(3) Phillips, D. *Creating Apps in Kivy: Mobile with Python*; " O'Reilly Media, Inc.," 2014.

(4) Andersson, J. C. *Learning Microsoft Azure*; " O'Reilly Media, Inc.," 2023.

(5) Grinberg, M. *Flask Web Development*; " O'Reilly Media, Inc.," 2018.

(6) Van Rossum, G.; Drake, F. L. Introduction To Python 3: Python Documentation Manual Part 1. CreateSpace 2009.

(7) Villán, A. F. *Mastering OpenCV 4 with Python: A Practical Guide Covering Topics from Image Processing, Augmented Reality to Deep Learning with OpenCV 4 and Python 3.7*; Packt Publishing Ltd, 2019.

(8) Weigert, M.; Schmidt, U.; Haase, R.; Sugawara, K.; Myers, G. Star-Convex Polyhedra for 3D Object Detection and Segmentation in Microscopy. In *Proceedings of the IEEE/CVF winter conference on applications of computer vision*; 2020; pp 3666–3673.

(9) Schmidt, U.; Weigert, M.; Broaddus, C.; Myers, G. Cell Detection with Star-Convex Polygons. In *Medical image computing and computer assisted intervention–MICCAI 2018: 21st international conference, Granada, Spain, September 16-20, 2018, proceedings, part II 11*; Springer, 2018; pp 265–273.

(10) Van der Walt, S.; Schönberger, J. L.; Nunez-Iglesias, J.; Boulogne, F.; Warner, J. D.; Yager, N.; Gouillart, E.; Yu, T. Scikit-Image: Image Processing in Python. *PeerJ* **2014**, *2*, e453.

(11) Bradski, G.; Kaehler, A. *Learning OpenCV: Computer Vision with the OpenCV Library*; " O'Reilly Media, Inc.," 2008.

(12) Nowak, P. M.; Wietecha-Posłuszny, R.; Pawliszyn, J. White Analytical Chemistry: An Approach to Reconcile the Principles of Green Analytical Chemistry and Functionality. *TrAC Trends in Analytical Chemistry* **2021**, *138*, 116223.
